# Polyurethane–Chitosan Composites for Tissue Engineering: Novel Strategies, Therapeutic Potential and Limitations

**DOI:** 10.3390/ma19194110

**Published:** 2026-09-25

**Authors:** Karolina Kędra, Marcin Sobczak

**Affiliations:** 1 Institute of Physical Chemistry, Polish Academy of Sciences, 44/52 Kasprzaka Str., 01-224 Warsaw, Poland; kkedra@ichf.edu.pl; 2 Department of Pharmaceutical Chemistry and Biomaterials, Faculty of Pharmacy, Medical University of Warsaw, Banacha 1 Str., 02-097 Warsaw, Poland

**Keywords:** biomedical polyurethane, biomedical chitosan, composite biomaterials, biomedical polyurethane–chitosan composites, tissue engineering, regenerative medicine

## Abstract

Polyurethane–chitosan (PU–CS) composites have emerged as promising biomaterials for tissue engineering because they combine the tunable mechanical properties, elasticity, processability, and degradation behavior of polyurethanes (PU) with the hydrophilicity, biological functionality, and antimicrobial properties of chitosan (CS). This review critically analyzes PU–CS composites with particular emphasis on the relationships between polymer composition, intermolecular interactions, phase organization, scaffold architecture, physicochemical properties, degradation, and biological performance. Particular attention is given to hydrogen bonding and other interfacial interactions governing composite organization, as well as to the effects of PU chemistry, CS molecular characteristics, PU/CS ratio, porosity, fiber organization, and fabrication strategy. Cross-study comparison indicates that no single PU/CS composition or scaffold architecture is universally optimal and that regenerative performance cannot be predicted from polymer ratio alone. Instead, composition must be considered together with hydration, mechanics, morphology, biodegradation kinetics, and tissue-specific requirements. Current evidence demonstrates promising cytocompatibility and regenerative potential of PU–CS systems, particularly in wound healing and selected bone, cardiovascular, and other tissue-engineering applications. However, the evidence base remains predominantly preclinical. Major barriers to translation include raw-material variability, insufficient standardization, limited long-term and large-animal data, manufacturing reproducibility, sterilization effects, scale-up, and regulatory complexity. Emerging approaches, including advanced biofabrication, computational modeling, and artificial intelligence, may further support PU–CS development, although their direct application to this material class remains limited. Overall, future progress will depend on shifting from descriptive optimization of individual scaffold properties toward reproducible, quantitatively characterized, and translationally oriented PU–CS biomaterial design. This review critically evaluates recent progress in PU–CS composite biomaterials by integrating evidence from fundamental polymer chemistry, materials engineering, biology, regenerative medicine, and translational biomaterials research.

## 1. Introduction

The continuous increase in life expectancy, together with the growing incidence of trauma, degenerative diseases, congenital abnormalities, cancer, diabetes, and chronic inflammatory disorders, has substantially increased the global demand for advanced regenerative therapies.

Conventional clinical interventions, including autografts, allografts, xenografts, metallic implants, and synthetic prostheses, have undoubtedly improved patient outcomes during the last few decades. However, each of these approaches is associated with significant biological, surgical, and economic limitations. Autologous grafts remain the clinical gold standard because of their excellent histocompatibility and osteogenic potential. However, their use is constrained by donor-site morbidity, limited tissue availability, prolonged surgical procedures, and postoperative complications. Similarly, allogeneic and xenogeneic grafts are limited by immunological incompatibility, pathogen transmission, unpredictable remodeling, and, in some cases, ethical considerations. Consequently, tissue engineering has therefore evolved into one of the most dynamic and interdisciplinary research areas, integrating materials science, polymer chemistry, cell biology, immunology, biomechanics, biomaterial technologies, and clinical medicine to create biomimetic environments capable of directing endogenous or transplanted cells toward functional tissue regeneration rather than passive defect filling [1,2,3,4,5,6,7,8].

One of the major reasons for the limited clinical success of many tissue-engineered constructs is the persistent gap between excellent laboratory performance and successful clinical implementation. Thousands of biomaterial formulations have demonstrated promising results in vitro, while only a very small proportion have progressed to clinical evaluation or commercialization. Instead, successful translation depends on the simultaneous optimization of multiple interconnected parameters, including reproducible polymer synthesis, scalable manufacturing, sterilization compatibility, long-term storage stability, regulatory compliance, economic feasibility, and clinically relevant validation [5,7,8].

The historical evolution of tissue engineering demonstrates that future progress will depend less on the discovery of entirely new biomaterials than on the rational integration of molecular design, hierarchical scaffold architecture, immune regulation, and translational manufacturability. In this context, PU-CS composites represent an attractive model system because they combine complementary mechanical and biological functions within a single hybrid platform. However, their true clinical potential can only be realized if scaffold development is guided by multidisciplinary design principles rather than by empirical optimization of isolated material properties [2,3,6].

The introduction of biodegradable polymeric scaffolds during the 1990s represented a fundamental turning point in biomaterials research. Unlike permanent implants, degradable polymers were designed to provide temporary structural support while gradually transferring mechanical loads to newly regenerated tissues. This concept significantly expanded the functional role of biomaterials, transforming scaffolds into dynamic templates that direct tissue regeneration. Synthetic polymers such as poly(lactic acid)/polylactide (PLA), poly(glycolic acid)/polyglycolide (PGA), lactide (LA) and glycolide (GA) copolymers (PLGA), poly(ε-caprolactone) (PCL), poly(ethylene glycol) (PEG), and aliphatic or cycloaliphatic PUs rapidly became dominant scaffold materials because their molecular structures could be tailored to achieve predictable degradation rates, mechanical properties, and processing characteristics. Nevertheless, despite their excellent engineering versatility, these polymers are often characterized by insufficient cell adhesion and limited capacity to regulate complex regenerative processes without additional biological modifications [3,4,5].

In parallel, naturally derived polymers—including collagen, gelatin, fibrin, hyaluronic acid, alginate, silk fibroin, and CS—attracted considerable attention because they inherently resemble major constituents of the extracellular matrix. These biomacromolecules possess excellent biocompatibility and often contain chemical functionalities that interact directly with cell-surface receptors, growth factors, and extracellular proteins. Consequently, natural polymers often promote superior cellular attachment, migration, proliferation, and differentiation compared with synthetic materials. However, their widespread application remains constrained by inadequate mechanical strength, rapid and often unpredictable degradation, batch-to-batch variability, limited processability, and insufficient long-term structural stability. These limitations illustrate a recurring dilemma in biomaterials science: materials exhibiting outstanding biological performance frequently possess inferior mechanical properties, whereas mechanically robust synthetic polymers generally lack sufficient biological activity [1,2,3,5].

Composite biomaterials, therefore, represent an evolutionary step in tissue engineering because they enable simultaneous optimization of mechanical integrity, biodegradation behavior, surface chemistry, biological signaling, antimicrobial properties, and manufacturing flexibility. Hybridization also allows independent control over multiple structural hierarchies, ranging from molecular interactions to macroscopic scaffold architecture [3,7,8].

Among synthetic polymers investigated for regenerative medicine, PUs occupy a unique position because few other polymeric systems offer comparable flexibility in macromolecular design while simultaneously maintaining excellent mechanical performance, processability, and biological adaptability. Unlike conventional linear polymers, segmented PUs are materials composed of alternating soft and hard domains connected through urethane linkages. This segmented architecture enables independent control of elasticity, stiffness, biodegradation behavior, hydrophilicity, crystallinity, and surface chemistry by altering the chemical compositions of polyols, diisocyanates, and chain extenders. Consequently, PUs should not be regarded as a single biomaterial but rather as a versatile molecular platform from which numerous tissue-specific materials can be rationally engineered.

Despite these remarkable advantages, PUs are not an ideal biomaterial. Most biomedical PUs remain biologically passive—lack cell-recognition capable of directly regulating adhesion, migration, or differentiation. Their relatively hydrophobic surfaces often lead to nonspecific protein adsorption, suboptimal cellular attachment, and occasionally unfavorable inflammatory responses. Furthermore, degradation behavior varies considerably depending on polymer chemistry. Polyester-based PUs generally exhibit hydrolytic degradation, whereas polyether-based materials demonstrate greater oxidative stability but may undergo enzyme-mediated degradation in vivo. Aromatic diisocyanates may generate degradation products associated with cytotoxicity, prompting increasing interest in aliphatic PUs and non-isocyanate polyurethane (NIPU) technologies. These observations illustrate a fundamental limitation of PUs: although they offer unparalleled engineering versatility, their biological performance often requires additional molecular or surface modifications to achieve optimal regenerative outcomes [4,7,9,10]. Consequently, contemporary PUs research has progressively shifted from optimizing mechanical performance toward engineering multifunctional bioactive materials. Blending PU with naturally derived polysaccharides has emerged as one of the most effective approaches for simultaneously improving hydrophilicity, biological recognition, degradation behavior, antimicrobial activity, and cellular interactions without compromising mechanical integrity. In particular, CS has attracted exceptional interest because its physicochemical characteristics complement many of PU’s intrinsic limitations, creating opportunities for synergistic composite biomaterials [4,7,10].

Among naturally derived polysaccharides investigated for tissue engineering, CS occupies a particularly distinctive position because it simultaneously functions as a structural biomaterial, a biologically active macromolecule, and an immunomodulatory regulator of tissue regeneration. Unlike many naturally occurring polymers that primarily serve as passive extracellular matrix analogs, CS possesses intrinsic physicochemical characteristics that can directly influence cellular behavior, microbial colonization, inflammatory responses, and wound healing. These multifunctional properties originate from its unique molecular architecture, consisting of randomly distributed β-(1→4)-linked D-glucosamine and N-acetyl-D-glucosamine units generated through the partial deacetylation of chitin, the second most abundant natural polysaccharide after cellulose. The presence of primary amino groups distinguishes CS from most naturally occurring polysaccharides. It provides exceptional opportunities for chemical modification, ionic interactions, hydrogen-bond formation, and electrostatic binding of proteins, nucleic acids, growth factors, and therapeutic molecules [1,2,3,5]. The biological performance of CS is governed primarily by three interconnected molecular parameters: degree of deacetylation (DD), molecular weight, and distribution of acetylated residues along the polymer chain. These structural characteristics determine protonation behavior, surface charge density, solubility, crystallinity, degradation kinetics, and interactions with proteins and cellular membranes. CS with a higher degree of deacetylation generally exhibits increased cationic charge density under physiological acidic conditions, thereby enhancing electrostatic interactions with negatively charged glycosaminoglycans, extracellular proteins, bacterial membranes, and cell surfaces. Conversely, molecular weight strongly influences viscosity, mechanical behavior, degradation rate, and tissue penetration. Importantly, these parameters rarely act independently; instead, they collectively regulate the biological identity of chitosan. This observation has profound implications for tissue engineering, as apparently similar CS-based scaffolds may exhibit substantially different biological responses due to subtle variations in molecular characteristics that are often insufficiently reported in the literature [1,3,5]. One of the most remarkable characteristics of CS is its ability to mimic selected biochemical functions of the native extracellular matrix. Owing to its structural similarities to naturally occurring glycosaminoglycans, chitosan provides a favorable microenvironment for cell attachment, migration, and matrix remodeling while also acting as a reservoir for bioactive molecules. The positively charged amino groups facilitate interactions with negatively charged growth factors, cytokines, proteoglycans, and extracellular proteins, thereby influencing their local concentration, stability, and biological availability. Rather than functioning exclusively as a passive structural scaffold, CS actively participates in regulating cell–matrix communication, which is increasingly recognized as a critical determinant of successful tissue regeneration. Consequently, CS-containing biomaterials frequently exhibit enhanced cellular infiltration, improved vascularization, accelerated wound closure, and more organized extracellular matrix deposition compared with many purely synthetic polymeric systems [3,5]. Despite these outstanding biological properties, CS alone exhibits several limitations that substantially restrict its clinical applicability. Native CS scaffolds generally possess poor tensile strength, relatively low elastic modulus, limited fatigue resistance, and insufficient dimensional stability under physiological conditions. Their degradation behavior is often highly variable and depends not only on molecular characteristics but also on local enzymatic activity, pH, ionic composition, and scaffold morphology. Furthermore, CS exhibits limited solubility at physiological pH and is typically processable only under mildly acidic conditions, complicating manufacturing and large-scale production. Considerable batch-to-batch variability arising from differences in biological sources, extraction methods, purification procedures, and deacetylation protocols further contributes to inconsistencies among published studies. These limitations are among the principal reasons why, despite decades of intensive investigation, relatively few CS-based biomaterials have achieved widespread clinical implementation [1,2,3,5,6,7].

Consequently, contemporary research has increasingly focused on integrating CS with mechanically robust synthetic polymers that compensate for its structural weaknesses while preserving its exceptional biological functionality. Among the various synthetic partners investigated—including PCL, PLA, PEG, and PLGA-PUs appear particularly attractive because their segmented molecular architecture provides mechanical adaptability, tunable degradation, and extensive hydrogen-bonding capability [6,7,8]. Importantly, the combination of PU and CS should not be interpreted simply as blending a synthetic polymer with a natural polysaccharide. Rather, it represents the convergence of two complementary design philosophies: mechanical engineering, embodied by PU, and biological engineering, embodied by CS. This complementarity forms the conceptual basis for the multifunctional PU-CS composite systems discussed throughout this review.

For many years, interest in PU-CS composites has been rapidly growing. Contemporary scaffold design is no longer driven by the search for a single “ideal polymer” but rather by the rational integration of complementary materials that collectively meet the numerous, often contradictory, requirements of tissue regeneration. Successful scaffolds must simultaneously provide mechanical stability, controlled biodegradation, cytocompatibility, vascularization, antimicrobial protection, immunomodulation, manufacturing reproducibility, and regulatory compatibility—objectives that rarely can be achieved by any individual polymeric system. Consequently, hybrid composite biomaterials have emerged not merely as combinations of existing materials but as integrated multifunctional platforms in which synergistic interactions generate biological and physicochemical properties that cannot be predicted solely from the characteristics of their individual constituents. Within this context, PU-CS composites represent one of the most promising examples of rational hybrid biomaterial design because they combine two fundamentally complementary material philosophies: engineering functionality and biological functionality [1,2,3,7,8]. Unlike conventional polymer blends, PU-CS composites should be interpreted as dynamic molecular systems in which extensive interfacial interactions fundamentally influence scaffold performance. Hydrogen bonding between urethane carbonyl groups and hydroxyl or amino functionalities of chitosan, electrostatic interactions, phase morphology, and nanoscale domain organization collectively determine not only mechanical behavior but also water uptake, swelling, degradation kinetics, protein adsorption, cellular attachment, and inflammatory responses. Consequently, the interface between PU and CS becomes an active structural component rather than merely the boundary separating two polymer phases [3,6]. From a biological perspective, PU-CS composites provide an opportunity to integrate two complementary regulatory systems within a single scaffold. PU predominantly controls the mechanical microenvironment by regulating elasticity, stress distribution, fatigue resistance, and biodegradation kinetics. In contrast, CS primarily modulates the biological microenvironment by influencing protein adsorption, antimicrobial activity, immune regulation, extracellular matrix remodeling, and cell–matrix communication. Importantly, these functions are not independent. Mechanical properties directly affect mechanotransduction pathways, while biological signals simultaneously regulate matrix synthesis and tissue remodeling. Consequently, regenerative outcomes emerge from continuous reciprocal interactions between mechanics and biology rather than from either factor alone [7,8]. The remarkable diversity of PU-CS composite architectures further expands their biomedical potential. Depending on fabrication strategy and intended clinical application, these materials may be processed into electrospun nanofibers, injectable hydrogels, cryogels, porous foams, multilayer membranes, vascular grafts, cardiac patches, wound dressings, nerve conduits, bioinks for three-dimensional bioprinting, and drug delivery systems (DDS). Such structural diversity enables independent control over pore size, fiber alignment, degradation behavior, mechanical anisotropy, and the spatial distribution of biological signals. Importantly, scaffold architecture increasingly appears to be as influential as chemical composition itself. Hierarchical organization at the nano-, micro-, and macroscales determines nutrient diffusion, vascular invasion, immune cell infiltration, and tissue maturation [1,2,3,5,6,7,8]. Despite their exceptional promise, PU-CS composites remain far from routine clinical implementation. Current research is characterized by considerable heterogeneity in PU chemistry, CS molecular characteristics, fabrication methods, crosslinking strategies, sterilization procedures, biological models, and evaluation protocols. As a consequence, direct comparison between published studies is frequently impossible, and mechanistic understanding remains fragmented. Moreover, most investigations focus on short-term cytocompatibility and isolated biological endpoints, whereas far fewer studies examine long-term degradation, immune modulation, large-animal validation, manufacturing scalability, or regulatory readiness. These limitations emphasize that future progress will require not only the synthesis of additional composite formulations but also the establishment of standardized design principles that systematically link polymer chemistry, processing, structure, biological performance, and translational feasibility [3,5,7,8].

PU-CS composites have attracted steadily increasing scientific attention over the past two decades, resulting in numerous experimental studies and several high-quality review articles. Existing reviews have substantially improved our understanding of PU chemistry, CS modification, composite fabrication techniques, hydrogel systems, electrospinning, wound healing applications, and selected tissue-specific engineering strategies. Nevertheless, most available reviews remain organized according to traditional descriptive classifications based on polymer type, fabrication method, or biomedical application. Consequently, they frequently summarize the existing literature without establishing generalizable principles that explain why certain composite formulations consistently outperform others despite apparently similar chemical compositions. More importantly, relatively little attention has been paid to integrating polymer chemistry and physics, mechanobiology, regenerative immunology, manufacturing science, and translational medicine into a unified conceptual framework to guide the rational development of clinically relevant composite biomaterials. A second important limitation of the current literature is the disproportionate emphasis on isolated experimental outcomes rather than on the mechanisms that govern regenerative success. Numerous publications report improvements in cell viability, proliferation, antibacterial activity, or mechanical strength following incorporation of chitosan into PU matrices. However, far fewer studies attempt to explain the molecular and biological mechanisms underlying these observations or to identify common principles linking polymer chemistry to long-term regenerative performance. Furthermore, variables such as phase morphology, interfacial hydrogen bonding, biodegradation kinetics, macrophage polarization, vascular remodeling, scaffold anisotropy, and manufacturing reproducibility are usually investigated independently despite their strong biological interdependence. A third challenge arises from the limited integration of translational considerations into biomaterials research. The overwhelming majority of published studies focus on demonstrating proof of concept in vitro or on short-term efficacy in small-animal models. In contrast, issues directly relevant to clinical implementation—including manufacturing scalability, sterilization compatibility, batch-to-batch reproducibility, quality control, regulatory approval, long-term safety, economic feasibility, and post-market surveillance—remain comparatively underrepresented. Consequently, remarkable scientific progress has not been accompanied by a proportional increase in clinically approved PU-CS biomaterials.

This review aimed to summarize existing knowledge about PU-CS composites used in tissue engineering. However, the main motivation was to discuss the identification of current research priorities and to provide practical guidance for the rational development of clinically translatable PU-CS composite biomaterials.

The literature search was designed to cover both the fundamental literature concerning biomedical polyurethanes (PU) and chitosan (CS) and, more specifically, experimental studies addressing PU-CS composites for tissue engineering and regenerative medicine. Searches were conducted using Web of Science Core Collection, Scopus, PubMed/MEDLINE, and Google Scholar. The principal search keywords included: “polyurethane chitosan composite”, “polyurethane-chitosan scaffold”, “polyurethane-chitosan tissue engineering”, “polyurethane-chitosan biomaterial”, “polyurethane-chitosan electrospinning”, “polyurethane-chitosan hydrogel”, “polyurethane-chitosan wound healing”, “polyurethane-chitosan bone tissue engineering”, “polyurethane-chitosan cartilage”, “polyurethane-chitosan nerve regeneration”, “polyurethane-chitosan vascular”, “biodegradable polyurethane-chitosan”, “waterborne polyurethane-chitosan”, “polyurethane-chitosan cytocompatibility”, “polyurethane-chitosan degradation”, and combinations of these terms with “mechanical properties”, “porosity”, “cell adhesion”, “biocompatibility”, “immune response”, “sterilization”, “scale-up”, “manufacturing”, and “clinical translation”.

The principal time window covered publications from 2000 to 2026, while earlier seminal studies were additionally included when necessary to describe the historical development, fundamental PU/CS chemistry, or mechanisms relevant to composite formation. Priority was given to peer-reviewed original research articles reporting experimentally characterized PU–CS materials. Review articles were used primarily to identify additional primary studies and to provide a broader background concerning PU and CS biomaterials.

Studies were included when they: (i) investigated PU–CS materials or provided directly relevant mechanistic information concerning PU or CS; (ii) reported sufficient information on composition, fabrication, physicochemical characterization, mechanical behavior, degradation, cytocompatibility, or biological performance; and/or (iii) addressed tissue-engineering, regenerative-medicine, wound-healing, or closely related biomedical applications. Studies were excluded when they concerned non-biomedical PU–CS applications, lacked sufficient experimental characterization, duplicated previously reported data, or provided no information relevant to the structure–property–biological-performance relationships discussed in this review. No formal meta-analysis was performed due to substantial heterogeneity across polymer chemistry, CS molecular characteristics, fabrication protocols, specimen geometry, mechanical-testing conditions, degradation protocols, cell models, and biological endpoints.

## 2. Biomedical Polyurethanes for Tissue Engineering

Nevertheless, this remarkable versatility has also contributed to considerable confusion within the literature. The term “PU” is frequently used as though it referred to a single material with well-defined physicochemical characteristics. In reality, biomedical PUs encompass an extensive family of chemically distinct polymers whose biological behavior may differ profoundly depending on molecular composition, phase morphology, degradation mechanism, and processing history. Consequently, comparisons between independent studies frequently become problematic because scaffolds described simply as “PU” often differ substantially in soft-segment chemistry, diisocyanate type, chain extenders, molecular weight distribution, crosslink density, and fabrication methodology. This lack of standardized reporting remains one of the principal barriers to establishing universal structure–property relationships for biomedical PUs [11,12,13,14,15,16,17,18,19,20,21].

Soft segments constitute the continuous phase of most segmented PUs and largely determine their flexibility, elasticity, resilience, and biodegradation behavior. These domains are commonly synthesized from oligomers (mainly oligomeric diols) such as PCL, PLA, PGA, PLGA, PEG, poly(propylene glycol) (PPG), poly(tetramethylene oxide) (PTMO), polycarbonate (e.g. poly(trimethylene carbonate), (PTMC)), poly(3-hydroxybutyrate) P(3HB), PCL-PEG, and PLA-PEG oligomers (Figure 1). Selection of the polyol chemistry directly influences macromolecular mobility, crystallinity, hydrophilicity, oxidative stability, hydrolytic susceptibility, and long-term mechanical behavior. Consequently, soft segments are the primary molecular components that tailor PU to the biomechanical requirements of specific tissues [11,12,13,14,15,16,17,18,19,20,21].

Polyester-based soft segments generally exhibit relatively rapid hydrolytic degradation because ester bonds are susceptible to cleavage under physiological conditions. This property makes polyester PUs particularly attractive for temporary regenerative scaffolds requiring gradual replacement by newly formed tissue. However, accelerated biodegradation may also lead to premature loss of mechanical support and the accumulation of acidic degradation products that can influence local inflammatory responses. In contrast, polyether-based PUs display superior hydrolytic stability and long-term flexibility. However, they may exhibit increased susceptibility to oxidative degradation mediated by reactive oxygen species released by activated inflammatory cells. Polycarbonate-based soft segments often provide an intermediate solution by combining enhanced oxidative resistance with more predictable degradation behavior and improved long-term mechanical stability. These observations illustrate that biodegradation kinetics should be regarded as an intrinsic consequence of molecular design rather than an isolated material property [10,14,19,20].

Whereas soft segments provide elasticity and molecular mobility, hard segments constitute the principal structural framework responsible for mechanical reinforcement and long-term dimensional stability of segmented PUs. These rigid domains are generated by reactions between diisocyanates and low-molecular-weight chain extenders, producing densely hydrogen-bonded urethane- and urea-rich regions with restricted molecular mobility. Segmented PUs derive much of their mechanical strength from reversible physical crosslinking, enabling simultaneous toughness, elasticity, fatigue resistance, and self-adjustment under cyclic mechanical loading. This dynamic nature of hard-segment organization distinguishes PUs from many conventional biodegradable polymers and contributes substantially to their unique suitability for mechanically demanding tissue-engineering applications [10,15,17,19].

The physicochemical behavior of hard segments is determined primarily by the chemical structure of the diisocyanate, the nature of the chain extender, and the resulting hydrogen-bonding network. Aromatic diisocyanates, including methylene diphenyl diisocyanate (MDI) and toluene diisocyanate (TDI), generally produce materials exhibiting superior mechanical strength because their rigid aromatic rings promote efficient molecular packing and stronger intermolecular interactions. However, degradation of aromatic PUs may generate aromatic amines associated with potential cytotoxicity and long-term safety concerns, limiting their application in biodegradable implantable scaffolds. Consequently, contemporary biomedical PUs increasingly employ aliphatic or cycloaliphatic diisocyanates such as 1,6-hexamethylene diisocyanate (HDI), lysine diisocyanate (LDI), isophorone diisocyanate (IPDI), or 1,4-diisocyanatobutane (BDI), which generally exhibit improved biocompatibility despite somewhat reduced rigidity (Figure 2) [9,10,11,12].

Chain extenders represent another critical yet frequently underestimated component of PU architecture. Although they contribute only a relatively small fraction of the polymer mass, chain extenders strongly influence hydrogen-bond density, crystallinity, hard-segment packing, and phase morphology. Commonly employed chain extenders include 1,4-butanediol (BDO), diethylene glycol, and various diamines. Their molecular geometry determines the spacing and orientation of urethane groups, thereby regulating the formation of ordered hydrogen-bonded nanodomains. Consequently, relatively minor modifications in chain-extender chemistry may produce substantial changes in tensile strength, elastic recovery, fatigue resistance, and degradation behavior. These observations emphasize that rational PUs engineering requires optimizing the entire molecular architecture rather than the isolated adjustment of individual polymer components [9,10,11,12,15,18].

Microphase separation represents one of the defining structural characteristics of segmented PUs and arguably the least appreciated determinant of their biological behavior. Because soft and hard segments differ substantially in polarity, flexibility, and intermolecular interactions, complete molecular mixing is thermodynamically unfavorable. Instead, segmented PUs spontaneously organize into nanoscale heterogeneous structures consisting of soft continuous domains interspersed with hard-segment-rich nanodomains. The extent of phase separation depends on numerous variables, including segment chemistry, molecular weight, hydrogen-bond density, processing conditions, thermal history, and solvent evaporation kinetics. Rather than representing an undesirable structural imperfection, controlled microphase separation is essential for achieving the remarkable combination of elasticity and mechanical strength characteristic of biomedical PUs [10,11,14,15].

A very important consequence of the interesting structure of PUs is the regulation of degradation behavior. Degradation should not be regarded simply as material disappearance but as a dynamic biological process that continuously modifies scaffold mechanics, permeability, surface chemistry and physics, and cellular interactions throughout tissue regeneration. Hydrolytic cleavage of ester bonds, oxidative degradation mediated by inflammatory cells, enzymatic activity, and mechanical fatigue occur simultaneously and interact with one another during implantation. Consequently, biodegradation kinetics influence not only scaffold longevity but also the temporal sequence of biological events, including vascularization, matrix deposition, and load transfer to newly formed tissue. Ideally, degradation should proceed synchronously with tissue regeneration, preserving sufficient mechanical support during early healing while gradually transferring functional loads to the regenerating tissue. Achieving such temporal synchronization remains one of the major challenges in contemporary PUs engineering [18,20,21].

### 2.1. Synthesis Strategies for Biomedical Polyurethanes

Among the numerous synthetic strategies developed for biomedical PUs, the prepolymer method remains the most widely employed because it provides excellent control over molecular architecture and minimizes undesirable side reactions (Figure 3). In this approach, an excess of diisocyanate initially reacts with hydroxyl-terminated polyols to generate isocyanate-terminated prepolymers, which are subsequently chain-extended using low-molecular-weight diols, diamines, or water. The prepolymer strategy has become the preferred approach for fabricating PU scaffolds intended for regenerative medicine [4,13,17,18].

Growing environmental awareness and increasingly stringent regulatory requirements have stimulated considerable interest in waterborne PUs (WPUs) as environmentally friendly alternatives to conventional solvent-based systems. Waterborne polyurethanes are synthesized by incorporating hydrophilic ionic or nonionic groups into PU chains, thereby enabling stable aqueous dispersions without requiring large quantities of volatile organic solvents. From a biomedical perspective, this strategy provides several important advantages, including reduced solvent toxicity, improved process safety, compatibility with biologically active molecules, and simplified fabrication of porous scaffolds, coatings, hydrogels, and electrospun fibers [4,13,18]. Importantly, waterborne PU technology also expands opportunities for composite fabrication with hydrophilic natural polymers such as CS. Conventional hydrophobic PU systems frequently require organic solvents incompatible with polysaccharides or biological molecules, whereas aqueous PU dispersions facilitate homogeneous polymer blending under considerably milder processing conditions. This compatibility significantly enhances interpolymer interactions, hydrogen-bond formation, and uniform distribution of biological components throughout the scaffold. Consequently, waterborne PU technology represents a particularly attractive platform for developing PU-CS composites discussed in subsequent sections of this review [9,10,11].

### 2.2. General Limitations of Biomedical Polyurethane Scaffolds

Despite their remarkable versatility, biomedical PUs remain subject to several important scientific and translational limitations, warranting the development of multifunctional composite systems. First, most PU formulations exhibit relatively limited intrinsic biological activity. Their regenerative potential, therefore, depends largely on indirect modulation of the biological microenvironment through physicochemical properties rather than active biochemical signaling [14,15,17,18,20,21]. Second, PUs’ biodegradation remains difficult to predict under physiological conditions because hydrolysis, oxidation, enzymatic activity, and mechanical fatigue occur simultaneously and influence one another throughout tissue remodeling. Standardized in vitro degradation protocols often fail to replicate these complex biological environments, leading to substantial discrepancies between laboratory observations and clinical behavior. This limitation complicates scaffold optimization and regulatory evaluation, particularly for long-term implantable devices [20,21]. Third, increasing regulatory expectations regarding reproducibility, sterilization, manufacturing consistency, extractables and leachables, long-term biocompatibility, and GMP production impose additional barriers to clinical translation. Many experimental PU scaffolds demonstrate excellent biological performance but remain unsuitable for commercialization because their synthesis, processing, or characterization cannot be reliably reproduced at an industrial scale [4,10,11,12,18,20].

Reassuring, PUs have evolved from a mechanically optimized engineering polymer into one of the most sophisticated molecular platforms available for regenerative medicine. Nevertheless, insufficient biological signaling, modest antimicrobial activity, incomplete immune regulation, and limited intrinsic bioactivity mean that PU alone is rarely sufficient to recapitulate the complexity of native regenerative microenvironments.

## 3. Biomedical Chitosan 

Although CS is frequently described as a single biomaterial, its biological performance is governed primarily by two interrelated molecular parameters: the degree of deacetylation (DD) and molecular weight. Together, these variables regulate protonation behavior, chain conformation, crystallinity, solubility, viscosity, enzymatic degradation, protein adsorption, and interactions with cells and extracellular matrix components [5,6,22]. The DD directly determines the density of primary amino groups available for protonation and intermolecular interactions. Increasing DD generally enhances positive surface charge under physiological acidic conditions, improving electrostatic interactions with negatively charged proteins, glycosaminoglycans, bacterial membranes, and cellular surfaces. Higher DD is frequently associated with improved antimicrobial activity, greater hemostatic potential, enhanced protein adsorption, and stronger interactions with extracellular matrix molecules. However, excessively high deacetylation may also increase crystallinity and reduce molecular flexibility, potentially influencing degradation kinetics and scaffold remodeling [5,20,21,22]. Molecular weight exerts equally profound effects on regenerative performance. High-molecular-weight CS generally exhibits superior mechanical stability, slower enzymatic degradation, increased viscosity, and prolonged structural persistence following implantation. Conversely, low-molecular-weight CS demonstrate enhanced aqueous solubility, improved tissue penetration, faster degradation, and frequently more pronounced biological signaling owing to greater molecular mobility. Importantly, these characteristics influence not only processing behavior but also inflammatory responses, angiogenesis, and extracellular matrix remodeling [2,3,5,6,22]. Increasing evidence suggests that DD and molecular weight also influence immune regulation. Nevertheless, published data remain partially contradictory because studies frequently employ CS preparations differing simultaneously in molecular weight, degree of deacetylation, purity, crystallinity, and fabrication methodology. As a result, establishing direct structure–immunology relationships remains difficult [2,6,20].

The physicochemical behavior of CS emerges from the complex interplay between molecular architecture, supramolecular organization, hydration, and environmental conditions. Unlike many synthetic polymers whose properties remain relatively stable across physiological environments, CS exhibits pronounced responsiveness to pH, ionic strength, enzymatic activity, and surrounding biological macromolecules. Such environmental sensitivity should not be regarded as a disadvantage but rather as an opportunity to design adaptive biomaterials that dynamically respond to the changing conditions encountered during tissue regeneration [2,6,22,23,24].

One of the main physicochemical properties of CS is its hydration behavior. Owing to the abundance of hydroxyl and amino groups, CS forms extensive hydrogen-bond networks with water molecules, generating hydrated interfacial layers that strongly influence protein adsorption, nutrient transport, diffusion of signaling molecules, and cellular interactions. The organization of these hydration layers regulates not only swelling but also molecular mobility, viscoelasticity, biodegradation kinetics, and scaffold permeability [2,20,22].

Swelling behavior further determines biological functionality by regulating pore expansion, oxygen diffusion, nutrient transport, and release of incorporated therapeutic molecules. Moderate swelling generally enhances cellular infiltration and vascularization, whereas excessive swelling may reduce mechanical stability and alter tissue geometry. Conversely, insufficient hydration may limit nutrient diffusion and delay tissue integration. Therefore, the optimization of swelling should be regarded as a balance between transport phenomena and structural integrity, rather than as a focus on maximizing water uptake. This principle is particularly important when CS is combined with PU, as the hydrophobic polyurethane phase can effectively mitigate excessive swelling while preserving biological functionality [3,6,22].

Surface charge represents another key physicochemical parameter governing the regenerative performance of CS. Positively charged amino groups facilitate the adsorption of negatively charged proteins and growth factors, while also contributing to antimicrobial activity by disrupting bacterial membranes. However, excessive surface charge may also alter protein conformation and influence immune recognition [3,5,6,20].

### 3.1. Biomedical Chitosan for Tissue Engineering

Although CS exhibits exceptional biological activity, its direct application in regenerative medicine remains limited by relatively poor solubility at physiological pH, modest mechanical strength, rapid swelling, limited long-term structural stability, and batch-dependent variability. These intrinsic limitations have stimulated the development of numerous chemical modification strategies designed to preserve the biological advantages of CS while extending its physicochemical functionality [5,6,22]. Chemical modification strategies can be broadly classified into three interconnected categories. The first includes substitution of amino or hydroxyl groups to alter solubility, charge density, and hydrogen-bonding capability. The second involves graft polymerization or conjugation with synthetic polymers and biologically active molecules to introduce new physicochemical or biological functions. The third encompasses crosslinking and network formation, enabling precise regulation of scaffold mechanics, degradation kinetics, and three-dimensional architecture [20,21,22,23,24,25,26,27,28,29,30,31,32,33,34,35,36]. Carboxymethyl chitosan (CMCS) is among the most extensively investigated CS derivatives because it substantially improves aqueous solubility under physiological conditions while maintaining excellent biocompatibility and biodegradability. The introduction of carboxymethyl groups reduces intermolecular hydrogen bonding, thereby limiting the solubility of native chitosan. It simultaneously introduces amphoteric behavior, broadening the polymer’s functional pH range. Consequently, CMCS has become particularly attractive for injectable hydrogels, wound dressings, drug-delivery systems, and tissue-engineering scaffolds requiring homogeneous aqueous processing [6,22,30]. Beyond improved processability, CMCS also influences biological performance. Enhanced hydration facilitates diffusion of nutrients, oxygen, signaling molecules, and degradation products throughout three-dimensional scaffolds. Moreover, the presence of additional carboxyl groups modifies protein adsorption and electrostatic interactions with extracellular matrix components, potentially influencing cellular adhesion and immune responses. Nevertheless, excessive substitution may reduce intermolecular cohesion and mechanical integrity, emphasizing that improved solubility should not be pursued independently of structural stability [2,3,5,30]. From the perspective of PU composites, CMCS offers additional advantages, as increased hydrophilicity improves compatibility with waterborne polyurethane dispersions and facilitates the formation of a homogeneous interpenetrating network [2,6,22,36].

The rapid development of biofabrication and additive manufacturing has stimulated increasing interest in methacrylated CS derivatives. The introduction of methacrylate groups enables rapid photocrosslinking under mild conditions compatible with living cells, thereby facilitating the fabrication of hydrogels, bioinks, and complex three-dimensional architectures through stereolithography and extrusion-based bioprinting [5,6,37,38]. Importantly, methacrylation expands processing versatility without eliminating the intrinsic biological activity of CS, allowing simultaneous control of scaffold geometry and biological performance [3,5,6,20,21,22]. Photocrosslinkable CS derivatives also provide unprecedented opportunities for integration with PU. Photocurable PU acrylates and methacrylated CS possess compatible polymerization chemistries that facilitate the formation of interpenetrating polymer networks exhibiting improved mechanical strength, structural fidelity, and printability. Such systems have become increasingly important in personalized regenerative medicine because they combine the architectural precision of additive manufacturing with the complementary physicochemical properties of synthetic and natural polymers [2,3,5,6,22].

### 3.2. General Limitations of Biomedical Chitosan Scaffolds

Despite its remarkable biological versatility, CS remains associated with several important limitations that continue to hinder widespread clinical translation. Perhaps the most significant challenge is the intrinsic variability of commercially available CS preparations. Differences in biological source, extraction procedures, purification protocols, degree of deacetylation, molecular weight, acetylation pattern, residual mineral content, endotoxin contamination, and batch-to-batch reproducibility frequently complicate comparison between experimental studies and impede regulatory standardization [2,22]. Mechanical performance represents another important limitation. Although CS exhibits excellent biological activity, its relatively low tensile strength, limited fatigue resistance, rapid swelling, and accelerated degradation restrict its use in applications requiring long-term structural support or physiological load transmission. These deficiencies become particularly evident in bone, cartilage, tendon, ligament, and cardiovascular tissue engineering. Accordingly, mechanical reinforcement via composite formation has become a dominant strategy in contemporary CS research [3,5,6,22].

## 4. Polyurethane–Chitosan Composites for Tissue Engineering

The convergence of PUs and CS represents one of the most significant developments in contemporary biomaterials science because it addresses a longstanding limitation of regenerative medicine: no single polymer simultaneously provides optimal mechanical performance, biological activity, controlled degradation, manufacturing versatility, and clinical translatability. PUs possess exceptional mechanical programmability, elasticity, fatigue resistance, and processability, but exhibit relatively limited intrinsic biological activity. Conversely, CS actively regulates immune responses, antimicrobial defense, angiogenesis, extracellular matrix remodeling, and cell–matrix interactions, yet lacks the mechanical robustness required for many load-bearing applications. Their combination, therefore, represents considerably more than the physical blending of two polymers. Instead, it creates an integrated multifunctional system in which complementary molecular characteristics interact across multiple components to generate biological functions that neither component can achieve independently (Figure 4) [1,7,8,33,39,40,41,42,43,44,45,46,47,48,49,50,51,52,53,54,55,56,57,58,59,60,61].

To date, several biomedical PU-CS composites with high application potential have been developed (Figure 5) (Table 1). The PU-CS composites were obtained by various methods.

Historically, most PU–CS composite studies have focused primarily on demonstrating improvements in isolated material properties, including increased hydrophilicity, enhanced cell adhesion, improved antibacterial activity, accelerated degradation, or superior mechanical performance. Although these studies collectively established the feasibility of combining the two polymers, they rarely attempted to explain the molecular mechanisms underlying the observed improvements. Consequently, current understanding remains largely descriptive, with relatively limited integration of polymer chemistry, supramolecular organization, mechanobiology, immunology, and tissue remodeling into a unified conceptual framework. This fragmentation of knowledge has likely slowed the rational development of next-generation composite biomaterials and contributed to persistent challenges in clinical translation [7,8,33,61].

The first level of organization responsible for the behavior of PU–CS composites is the molecular interface formed immediately after polymer blending. These hydrogen bonds are dynamic rather than static, continuously dissociating and reforming during hydration, deformation, biodegradation, and tissue remodeling. Consequently, they function simultaneously as structural stabilizers and adaptive molecular regulators that respond to changing physiological conditions [8,40,52,61].

Cross-study comparison indicates that no single PU/CS ratio or scaffold architecture is universally optimal. Increasing CS content generally enhances hydrophilicity, biological recognition, swelling, and susceptibility to enzymatic degradation, but excessive CS content may compromise mechanical integrity or induce macroscopic phase separation. Conversely, PU-rich compositions preserve elasticity, toughness, and dimensional stability but may show less favorable hydration and cellular interaction. The most successful systems, therefore, appear to operate within a composition–structure window in which interpolymer hydrogen bonding is sufficient to stabilize the interface without eliminating the dynamic phase organization required for hydration, deformation, and degradation. Importantly, fabrication architecture often modifies these relationships as strongly as composition: aligned electrospun systems favor anisotropic cell organization, microporous hydrogels facilitate infiltration and fluid transport, whereas double-network PU–CS bioinks enable unusually soft mechanical environments compatible with neural differentiation.

Quantitative comparison of representative PU–CS tissue-engineering systems reveals that scaffold architecture frequently modifies biological performance as strongly as bulk polymer composition. In electrospun systems, increasing CS content typically reduces fiber diameter and increases hydrophilicity, but excessive CS content may compromise mechanical integrity or processing stability. Aligned architectures introduce an additional variable by independently directing cell orientation from chemical composition. In hydrogel and microporous systems, large interconnected pores improve cell infiltration and fluid transport, whereas increased crosslinking generally enhances structural stability at the expense of permeability and degradation. Consequently, PU/CS ratio alone cannot predict regenerative performance; composition must be interpreted together with scaffold architecture, hydration, mechanics, and degradation kinetics.

Beyond improving compatibility between otherwise chemically distinct polymers, hydrogen bonding also regulates molecular mobility and phase organization. Increasing interpolymer hydrogen-bond density generally reduces macroscopic phase separation, enhances stress transfer between polymer domains, and improves structural homogeneity. However, excessive intermolecular association may also reduce chain mobility, increase brittleness, or restrict water diffusion. Consequently, optimal PU-CS composite performance depends on balancing intermolecular cohesion with sufficient molecular flexibility to preserve elasticity and facilitate dynamic biological interactions. This balance represents one of the fundamental molecular design principles governing PU–CS composites [1,7,8,61]. Electrostatic interactions further complement hydrogen bonding. Under mildly acidic conditions, protonated amino groups of CS interact with polar urethane domains, hydration shells, adsorbed proteins, and biologically active ions, creating highly dynamic interfacial environments. Although these electrostatic interactions are generally weaker than covalent bonds, they contribute significantly to hydration, protein adsorption, growth-factor retention, and cellular recognition. Importantly, hydrogen bonding and electrostatic interactions do not act independently but cooperate to establish adaptive supramolecular interfaces that continuously respond to physiological stimuli [8,33,61].

The molecular interface also determines the initial organization of water molecules within the composite. Hydrophilic CS domains attract extensive hydration layers, whereas PU contributes structural stability by limiting excessive swelling. As a consequence, PU–CS composites frequently exhibit balanced hydration characteristics that simultaneously facilitate nutrient transport, preserve mechanical integrity, and maintain favorable protein conformations [1,7].

The relationship between PU–CS intermolecular interactions and biological performance should be interpreted as a multistep rather than a direct causal process. Hydrogen bonding between PU urethane/urea groups and CS hydroxyl/amino groups modifies polymer miscibility, chain mobility, phase separation, surface exposure of CS-rich domains, hydration, and mechanical behavior. These physicochemical changes subsequently determine the amount, composition, conformation, and spatial distribution of adsorbed proteins. Because integrin-mediated cell adhesion occurs predominantly through this protein-conditioned interface rather than through direct contact with chains, changes in PU–CS supramolecular organization can ultimately influence adhesion formation, spreading, proliferation, migration, and differentiation. Thus, hydrogen bonding should be regarded as an upstream molecular regulator of the biological interface rather than as a direct biological stimulus.

Although polyurethane–chitosan composites are often described as simple polymer blends, their internal organization is considerably more complex. Rather than existing as completely homogeneous systems or fully separated phases, most composites exhibit partially compatible multiphase architectures in which PU-rich and CS-rich domains remain interconnected through dynamic intermolecular interactions. Consequently, the physicochemical and biological properties of these composites emerge primarily from interfacial organization rather than from the intrinsic properties of either polymer individually [1,7].

The morphology of PU–CS composites depends on numerous interconnected factors, including PU soft-segment chemistry, hard-segment content, CS molecular weight, degree of deacetylation, polymer ratio, solvent system, fabrication methodology, drying kinetics, and crosslinking strategy. These variables collectively determine domain size, interfacial thickness, hydrogen-bond density, crystallinity, water distribution, and pore organization. Importantly, relatively small modifications introduced during synthesis frequently propagate across multiple structural levels, producing disproportionately large changes in biological behavior. Such nonlinear relationships illustrate that composite performance cannot be predicted solely from bulk composition but requires a detailed understanding of hierarchical structural organization [8,33,39,40,43,61].

One of the most significant consequences of supramolecular organization is the modification of PU microphase separation. In conventional segmented polyurethane, thermodynamic incompatibility between soft and hard segments generates nanophase-separated structures responsible for elasticity and mechanical strength. Incorporation of CS partially reorganizes this morphology by introducing additional hydrogen-bonding sites that compete with polyurethane–polyurethane interactions. Depending on composition and processing conditions, this may reduce excessive hard-domain aggregation, increase interfacial compatibility, or induce secondary phase separation when polymer compatibility becomes insufficient. Thus, CS functions not merely as a reinforcing filler but as an active regulator of PU nanostructure [1,7,61].

Water distribution within the composite provides another important example of supramolecular regulation. Hydrophilic CS domains attract and organize hydration shells, whereas polyurethane limits unrestricted swelling by providing mechanically stable hydrophobic regions. As a consequence, water is not distributed uniformly throughout the scaffold but becomes compartmentalized within a heterogeneous microenvironment. This heterogeneous hydration influences diffusion of nutrients, oxygen, cytokines, enzymes, degradation products, and therapeutic molecules while simultaneously regulating local mechanical properties and protein adsorption. Accordingly, hydration should be regarded as a structural component of composite morphology rather than merely a consequence of water uptake [1,8,33].

The terms “synergy” and “emergent behavior” should be applied cautiously to PU–CS composites. In many published studies, the composite is compared only with a singleparent polymer or with a formulation that differs simultaneously in multiple parameters. Such experimental designs demonstrate improved performance but do not necessarily prove true synergy. Strict demonstration of synergy requires comparison of PU alone, CS alone, and the PU–CS composite under otherwise equivalent conditions, ideally accompanied by quantitative analysis showing that the composite response exceeds the expected additive contribution of the individual components. Accordingly, in this review, improvements supported by direct controls are described as experimentally demonstrated effects. In contrast, mechanistic interpretations based on hydrogen bonding, phase organization, hydration, and interfacial restructuring are referred to as proposed cooperative or emergent mechanisms unless direct experimental evidence is available.

Evidence directly connecting PU–CS hydrogen-bond density to macrophage phenotype remains limited. A mechanistically stronger interpretation is that PU–CS interactions regulate intermediate physicochemical variables—including surface chemistry or physics, protein adsorption, stiffness, topography, degradation rate, and degradation-product exposure—which are independently known to influence macrophage adhesion and activation. Simultaneously, CS can contribute intrinsic immunomodulatory activity. Therefore, the immune response to PU–CS scaffolds should be interpreted as an emergent consequence of composition, supramolecular organization, degradation, and scaffold architecture rather than as the result of a single molecular interaction.

An important limitation of many current biomaterials studies is the implicit assumption that scaffold properties remain constant after implantation. In reality, PU–CS composites participate in continuous bidirectional communication with surrounding tissues. Protein adsorption alters surface chemistry; immune-cell activity modifies degradation; biodegradation changes mechanical properties; evolving mechanics regulate mechanotransduction; extracellular matrix deposition progressively replaces scaffold material; and newly formed tissue subsequently modifies the mechanical loading experienced by the remaining scaffold. These feedback loops collectively create a phenomenon known in developmental biology as dynamic reciprocity, in which cells continuously remodel their extracellular environment while simultaneously responding to those modifications [1,33,42].

This concept fundamentally changes scaffold evaluation. Traditional biomaterials research frequently characterizes mechanics, degradation, swelling, or cytocompatibility at isolated experimental time points. However, such static measurements cannot adequately describe biomaterials whose physicochemical and biological characteristics continuously evolve during implantation. Future investigations should therefore increasingly employ longitudinal experimental approaches integrating real-time imaging, multiscale mechanical characterization, proteomics, metabolomics, spatial transcriptomics, and computational modeling to capture the temporal evolution of regenerative biomaterials [8,40,43,60,61].

### General Limitations of Polyurethane-Chitosan Composite Scaffolds

Despite extensive preclinical investigation, PU–CS composite scaffolds remain at an early translational stage (Figure 6). At present, the authors found no convincing evidence that a biodegradable PU–CS tissue-engineering scaffold comparable to those reviewed here has entered routine clinical use. Likewise, the available literature is dominated by in vitro studies and small-animal experiments rather than controlled human trials. This situation contrasts with the independent clinical histories of PU and CS: several PU formulations have long been used in medical devices. At the same time, selected CS-based technologies have undergone clinical evaluation. These precedents demonstrate the translational feasibility of the individual polymer classes but do not constitute evidence of clinical validation for their combination. The principal translational bottleneck is not a single biological deficiency but the accumulation of uncertainties as material complexity increases. PU introduces variability in soft/hard segment chemistry, catalysts, residual isocyanates, molecular weight, and degradation products (Table 2).

In contrast, CS introduces source-dependent variability in molecular weight, degree of deacetylation, purity, and endotoxin content. Composite formation introduces an additional level of variability in PU/CS ratio, phase morphology, interfacial hydrogen bonding, and pore architecture. Sterilization may further alter molecular weight, oxidation, hydrogen-bond organization, and mechanics.

Consequently, a formulation that performs well as a laboratory prototype may no longer exhibit identical properties after scale-up, sterilization, storage, and GMP manufacture.

One of the principal scientific limitations hindering clinical translation is the absence of predictive relationships linking molecular design with long-term biological performance. Most published studies report improvements in isolated experimental parameters—including cell viability, mechanical strength, antibacterial activity, or biodegradation—but rarely establish mechanistic connections among molecular interactions, supramolecular organization, immune regulation, mechanotransduction, and tissue remodeling. As a consequence, scaffold development often remains empirical, relying on iterative optimization rather than predictive biomaterial engineering [8,33,51].

Another important limitation concerns the widespread reliance on simplified experimental models. Conventional static cell cultures fail to recapitulate the complexity of living tissues, in which cells experience dynamic mechanical loading, heterogeneous oxygen gradients, immune-cell interactions, vascular remodeling, extracellular matrix turnover, and continuously changing biochemical environments. Similarly, many small-animal models differ substantially from human physiology in immune responses, tissue dimensions, biomechanics, and regenerative capacity. Consequently, encouraging preclinical results frequently overestimate clinical performance [7,8,33,51,61]. Perhaps the most overlooked scientific barrier involves insufficient characterization of the host response over time. Biomaterial evaluation often terminates after several weeks, even though clinically relevant remodeling of connective tissues, cartilage, bone, or cardiovascular structures may continue for many months or even years [7,61].

From a translational perspective, these barriers should not be considered equivalent or independent. Reproducibility is arguably the first problem that must be solved because an incompletely defined material cannot be meaningfully validated for sterilization, long-term safety, regulatory compliance, or clinical efficacy. The principal priority should therefore be establishing a material-specific critical quality attribute (CQA) framework. For CS, minimum CQAs should include molecular weight and distribution, degree and pattern of deacetylation, purity, endotoxin level, moisture content, and biological source. For PU, they should include monomer identity, soft/hard segment ratio, molecular weight distribution, residual monomer and catalyst content, phase morphology, and degradation product profile. At the composite level, PU/CS ratio, spatial distribution of both phases, hydrogen-bond organization, porosity, pore-size distribution, mechanical properties, swelling, and degradation kinetics should be defined within validated acceptance ranges.

Sterilization represents the second major priority. Sterilization cannot be selected solely after scaffold optimization because gamma irradiation, electron-beam treatment, ethylene oxide, plasma, steam, and supercritical CO_2_ can affect PU oxidation, chain scission/crosslinking, CS molecular weight, intermolecular hydrogen bonding, phase morphology, mechanical performance, and biological activity in different ways. Therefore, physicochemical and biological characterization should be performed on the final sterilized device rather than solely on the non-sterile laboratory prototype.

Scalability represents a third interconnected barrier. Methods that perform well on a laboratory scale may not preserve polymer mixing, solvent-removal kinetics, fiber morphology, pore architecture, or phase separation during industrial production. Electrospinning is a representative example: environmental conditions, flow rate, electric-field geometry, and collector configuration influence fiber diameter and porosity, and must therefore become controlled manufacturing parameters rather than laboratory variables. Waterborne PU technologies and continuous or automated fabrication processes may provide particularly attractive routes toward scalable PU–CS production.

Long-term biological safety constitutes an equally critical evidence gap. Most published PU–CS studies emphasize short-term cytocompatibility, whereas clinically relevant implants may remain in contact with tissues for months or years. Future studies should therefore determine not only cell viability but also chronic inflammatory and foreign-body responses, macrophage phenotypes, fibrous encapsulation, systemic distribution and clearance of degradation products, genotoxicity when relevant, and the temporal relationship between scaffold degradation and tissue regeneration. Large-animal studies under clinically realistic mechanical and anatomical conditions will be necessary before laboratory success can be reliably extrapolated to humans.

Finally, regulatory strategy should be integrated into biomaterial development from the earliest design stage. Increasing the functional complexity of PU–CS systems by incorporating drugs, growth factors, living cells, or nanoparticles may improve therapeutic performance but simultaneously increase the manufacturing and regulatory burden. Therefore, the clinically most successful PU–CS scaffold may not necessarily be the formulation exhibiting the highest number of biological functions, but rather the simplest formulation capable of providing a clinically meaningful regenerative benefit with reproducible manufacture, predictable degradation, and an acceptable safety profile.

Translation of PU–CS composites should be treated as a sequential decision process rather than as an endpoint reached after successful in vitro testing. The first requirement is the standardization of both raw materials. For PU, this includes defined soft- and hard-segment chemistry, molecular weight distribution, residual monomers, catalyst content, and degradation product profile. For CS, molecular weight, degree of deacetylation, purity, biological source, endotoxin content, and moisture must be controlled. Only after these parameters have been defined should a reproducible PU/CS ratio, phase morphology, and scaffold architecture be established.

The second stage requires tissue-specific characterization of mechanical properties, porosity, swelling, transport behavior, and degradation. Biological screening should subsequently compare the composite with appropriate controls, ideally including PU alone and CS alone, rather than relying only on untreated-cell controls.

Promising formulations should then progress from conventional cytocompatibility studies to mechanistic evaluation of protein adsorption, immune responses, degradation products, and tissue-specific functional endpoints. Small-animal proof-of-concept studies should be followed by long-term and, where relevant, large-animal models that reproduce clinically relevant scaffold dimensions, mechanical loading, and implantation conditions.

Importantly, scale-up and sterilization should be introduced before final preclinical validation, rather than after. The material evaluated in pivotal safety studies should closely correspond to the final manufactured and sterilized product. Critical quality attributes should include PU/CS composition, molecular characteristics, phase morphology, residual chemicals, porosity, mechanical properties, swelling, degradation, and biological activity.

Regulatory strategy should be incorporated early because the classification and required evidence may differ substantially among an acellular scaffold, a drug-releasing PU–CS system, and a cell-containing biofabricated construct. Only after reproducible GMP-compatible production, validated sterilization, long-term safety, and clinically relevant efficacy have been established should human evaluation be initiated.

## 5. Future Perspectives

The evolution of PU–CS composites reflects a broader transformation occurring throughout regenerative medicine. Historically, biomaterials were designed primarily as passive structural substitutes intended to replace damaged tissues until natural healing occurred. Subsequent generations of scaffolds incorporated controlled biodegradation, improved mechanical properties, and enhanced biocompatibility. Contemporary biomaterials increasingly serve as biologically active platforms that regulate cellular behavior, immune responses, vascularization, and extracellular matrix remodeling. However, current evidence suggests that the next generation of regenerative materials will move even further toward adaptive biomaterials that continuously sense, interpret, and respond to dynamic physiological environments throughout tissue repair. Rather than optimizing isolated scaffold properties such as tensile strength or porosity, future PU–CS composites are expected to function as dynamic regenerative systems integrating mechanics, immunology, molecular sensing, and personalized medicine. This conceptual transition fundamentally changes biomaterial engineering, as scaffold performance is defined by its capacity to coordinate biological processes rather than merely preserve structural integrity. Consequently, future scaffold design should increasingly resemble systems engineering rather than conventional polymer science.

One of the most promising directions for PU–CS composites is the development of intelligent biomaterials that dynamically modify their properties in response to environmental signals. Extracellular matrix continuously adapts to alterations in mechanical loading, inflammation, oxygen availability, enzymatic activity, and biochemical signaling. In contrast, most currently available biomaterials remain largely static following implantation. Future PU-SC scaffold systems should increasingly reproduce this adaptive behavior through reversible molecular interactions, stimuli-responsive polymers, programmable biodegradation, and dynamic modulation of biological signaling.

Potential physiological stimuli include alterations in pH, oxidative stress, inflammatory cytokines, enzymatic activity, temperature, glucose concentration, mechanical loading, electrical stimulation, and local oxygen tension. PUs contribute tunable mechanical responsiveness, whereas CS provides environmentally sensitive protonation behavior and enzymatically regulated biodegradation. Integration of these complementary characteristics offers opportunities to develop scaffolds capable of autonomously modulating stiffness, permeability, degradation rate, drug release, or biological signaling in response to the evolving regenerative microenvironment.

Three-dimensional printing has already transformed scaffold fabrication through precise spatial control of architecture. The emerging field of four-dimensional (4D) bioprinting extends this concept by introducing the temporal dimension, enabling printed constructs to modify their geometry, mechanical properties, or biological activity following implantation in response to environmental stimuli. PU–CS composites appear particularly well suited for 4D biofabrication because reversible hydrogen-bond networks, hydration-dependent swelling, programmable biodegradation, and mechanically responsive PU domains collectively provide mechanisms for dynamic structural adaptation. Potential applications include self-folding vascular grafts, adaptive cartilage scaffolds, shape-memory wound dressings, mechanically responsive tendon implants, and self-adjusting nerve guidance conduits. Such technologies may fundamentally change surgical implantation by enabling biomaterials to undergo controlled structural transformation directly within the patient.

One of the most significant conceptual advances in regenerative medicine has been recognition that immune regulation represents an essential determinant of tissue repair rather than an undesirable side effect of biomaterial implantation. Future PU–CS composites should therefore be designed according to the principles of immunoengineering, in which scaffold chemistry, mechanics, biodegradation, and architecture are optimized to regulate innate and adaptive immune responses.

Rapid advances in genomics, transcriptomics, proteomics, metabolomics, glycomics, lipidomics, and spatial biology are expected to transform regenerative biomaterials by enabling unprecedented characterization of tissue responses to implanted scaffolds. Rather than relying exclusively on histological evaluation and selected biomarkers, future studies should integrate comprehensive molecular profiling throughout the regenerative process. Integration of multi-omics data with advanced computational analysis will permit identification of molecular pathways governing scaffold integration, macrophage polarization, angiogenesis, extracellular matrix remodeling, and long-term tissue maturation. Such datasets may ultimately facilitate predictive biomaterial engineering, in which scaffold composition is selected based on desired biological response profiles rather than on empirical optimization.

Artificial intelligence and machine learning should currently be considered emerging enabling technologies for PU–CS biomaterial development rather than established PU–CS fabrication strategies. Although AI-assisted material discovery, process optimization, image-based scaffold analysis, and predictive modeling are rapidly expanding across biomaterials science, direct experimental applications to PU–CS composites remain limited. Therefore, AI-related concepts discussed in this review should primarily be interpreted as future opportunities. Potential applications include predicting PU/CS composition–property relationships, optimizing electrospinning or biofabrication parameters, integrating mechanical and biological datasets, and selecting patient-specific scaffold architectures. Validation using experimentally generated PU–CS datasets will be required before such approaches can be considered established design tools for this material class.

Future development of PU–CS composites will also be influenced by increasing emphasis on sustainability and environmentally responsible manufacturing. CS already represents an excellent example of biomass valorization by converting marine by-products into high-value biomedical materials. Simultaneously, considerable progress is being achieved in the synthesis of bio-based PUs derived from renewable polyols, vegetable oils, lignin, castor oil, and carbohydrate-based feedstocks. The convergence of renewable PU chemistry and naturally derived CS creates opportunities to design regenerative biomaterials that simultaneously meet clinical, environmental, and economic sustainability criteria.

The future of PU–CS composites will not be determined primarily by the discovery of new polymers. Instead, progress will depend upon the successful integration of polymer chemistry, supramolecular science, immunology, mechanobiology, computational modeling, artificial intelligence, biofabrication, sustainability, manufacturing science, and clinical medicine into a unified interdisciplinary framework. Such integration represents a transition from designing better materials toward designing better regenerative systems.

## 6. Conclusions and Outlook

PU–CS composites should no longer be regarded as conventional polymer blends designed merely to combine the favorable properties of two biomaterials. Instead, the available evidence supports their classification as hierarchically organized adaptive regenerative systems, whose biological performance emerges from continuous interactions occurring across multiple structural and temporal scales. Molecular interface engineering, supramolecular organization, scaffold architecture, physicochemical adaptation, immune regulation, mechanobiological signaling, extracellular matrix remodeling, and tissue regeneration together constitute an integrated network that ultimately determines clinical performance. Consequently, successful scaffold design requires simultaneous optimization of these interconnected processes rather than isolated improvement of individual material characteristics.

Despite remarkable progress achieved during the past two decades, several important scientific questions remain unresolved. Quantitative relationships between molecular interactions and long-term regenerative outcomes remain poorly understood. Predictive computational models linking PU chemistry, CS molecular architecture, supramolecular morphology, biodegradation kinetics, immune regulation, and tissue remodeling have yet to be established. Furthermore, standardized descriptors for objectively comparing PU–CS composites across laboratories are still lacking. Addressing these limitations will require substantially greater integration of polymer chemistry, mechanobiology, computational modeling, systems immunology, and quantitative biomaterials science. Another major challenge concerns the limited availability of long-term translational evidence. Although numerous studies report excellent cytocompatibility and encouraging small-animal outcomes, relatively few investigations evaluate clinically relevant large-animal models, long-term scaffold remodeling, functional tissue recovery, or regulatory readiness. This imbalance between fundamental research and translational validation contributes significantly to the persistent gap separating laboratory innovation from routine clinical practice.

Clinical translation of PU–CS composites will depend not only on continued improvements in material performance but equally on successful integration of manufacturing science, regulatory strategy, industrial scalability, health economics, and clinical evidence generation. Future regenerative PU-CS biomaterials must therefore be designed with simultaneous consideration of physicochemical performance, biological functionality, manufacturability, sterilization, quality assurance, regulatory compliance, and cost-effectiveness.

In summary, PU–CS composites represent far more than a combination of a mechanically robust synthetic polymer and a biologically active natural polysaccharide. They constitute a new class of hierarchically organized adaptive biomaterials whose performance emerges from coordinated interactions extending from molecular chemistry to clinical tissue regeneration. Their future success will depend less on incremental optimization of individual scaffold properties than on the development of predictive design strategies integrating polymer science, supramolecular chemistry, mechanobiology, immunology, computational modeling, advanced manufacturing, and translational medicine. The next generation of polyurethane–chitosan biomaterials should account for molecular interface engineering, dynamic supramolecular organization, adaptive scaffold architecture, controlled immune modulation, synchronized tissue remodeling, and translational readiness. Adoption of this framework may accelerate the development of clinically relevant regenerative PU-CS biomaterials.

## Figures and Tables

**Figure 1:**
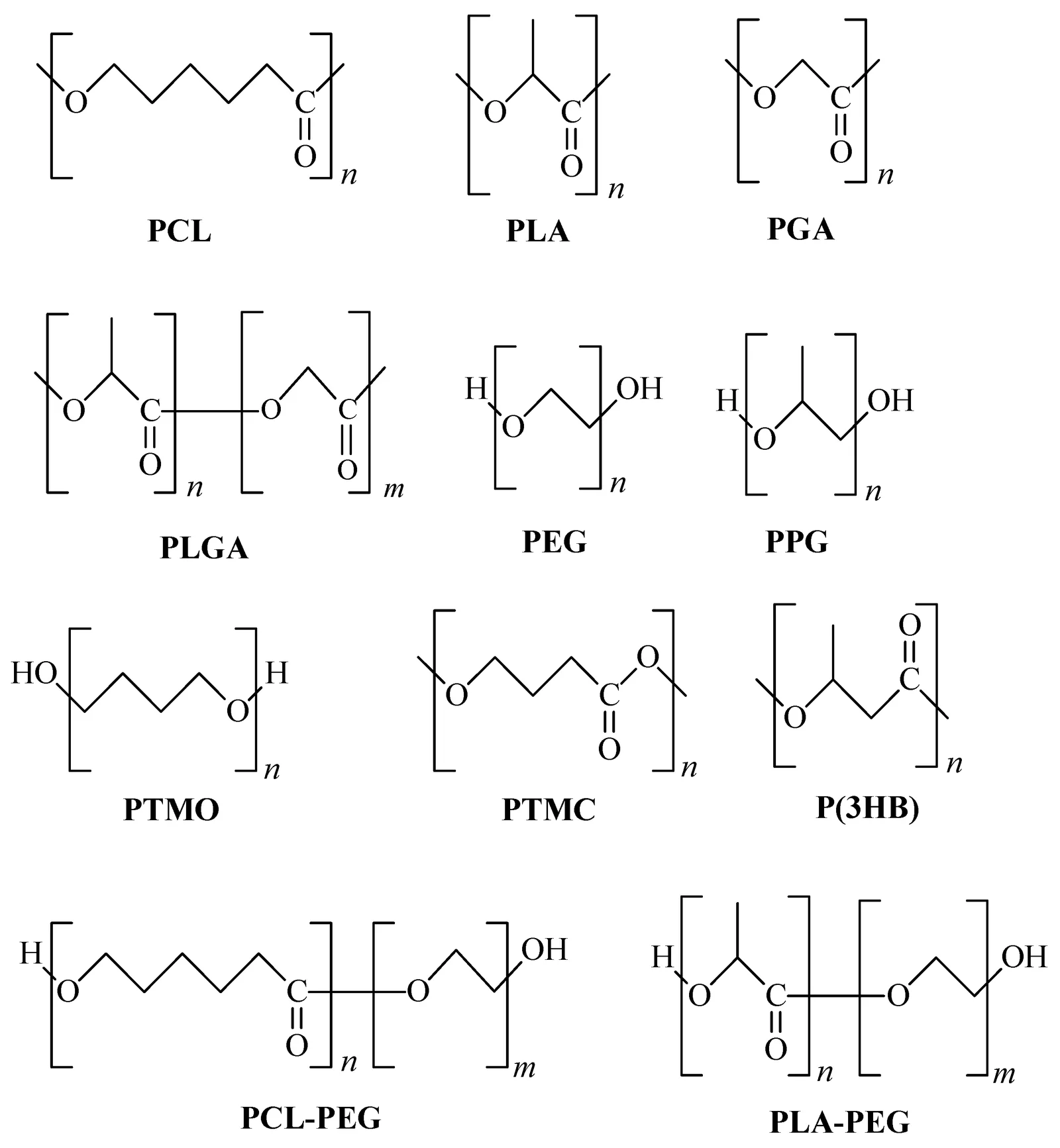
Oligomers used in the synthesis of biomedical polyurethanes (applied in tissue engineering).

**Figure 2:**
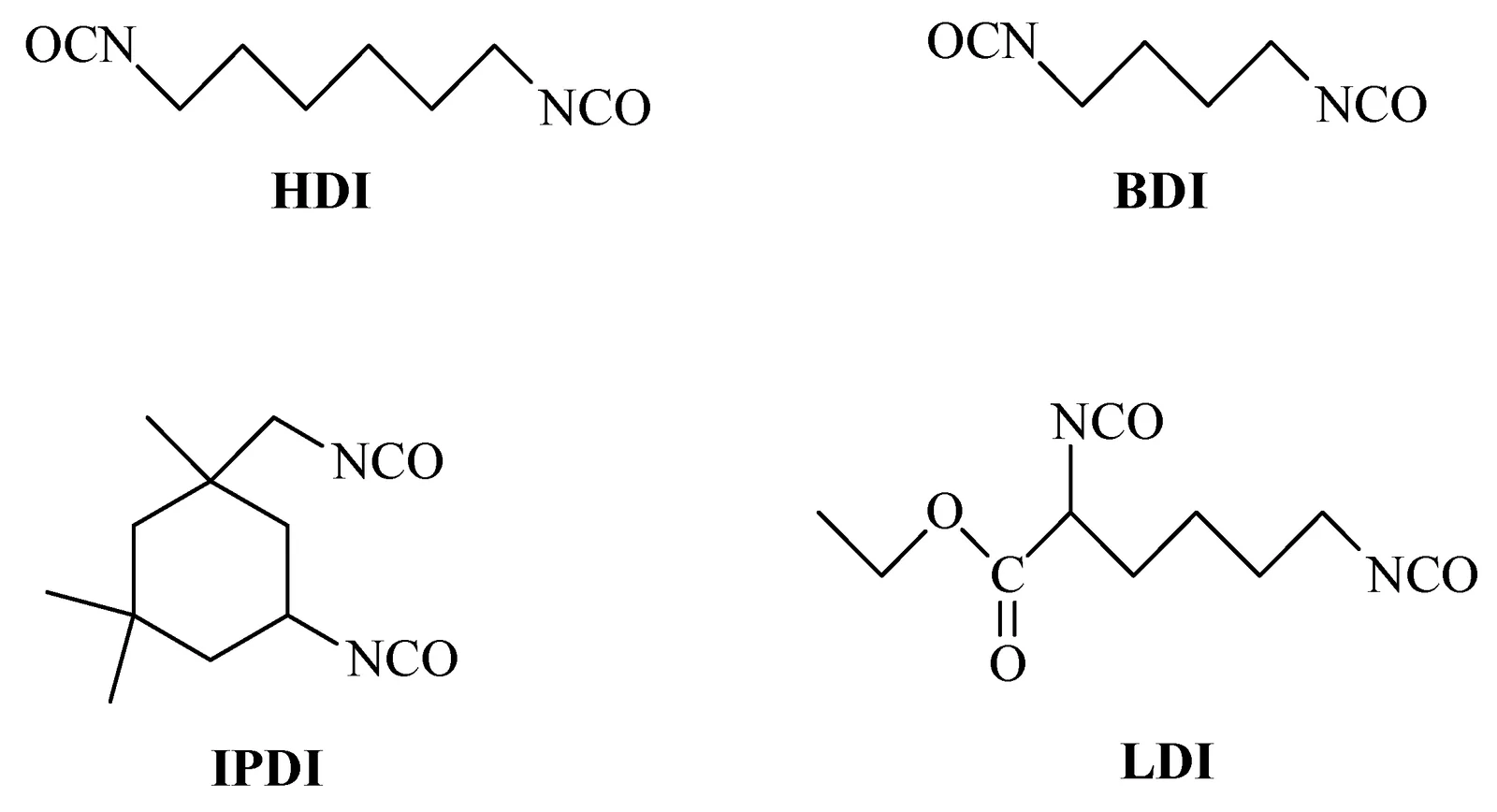
Aliphatic or cycloaliphatic diisocyanates used in biomedical polyurethane synthesis (applied in tissue engineering).

**Figure 3:**
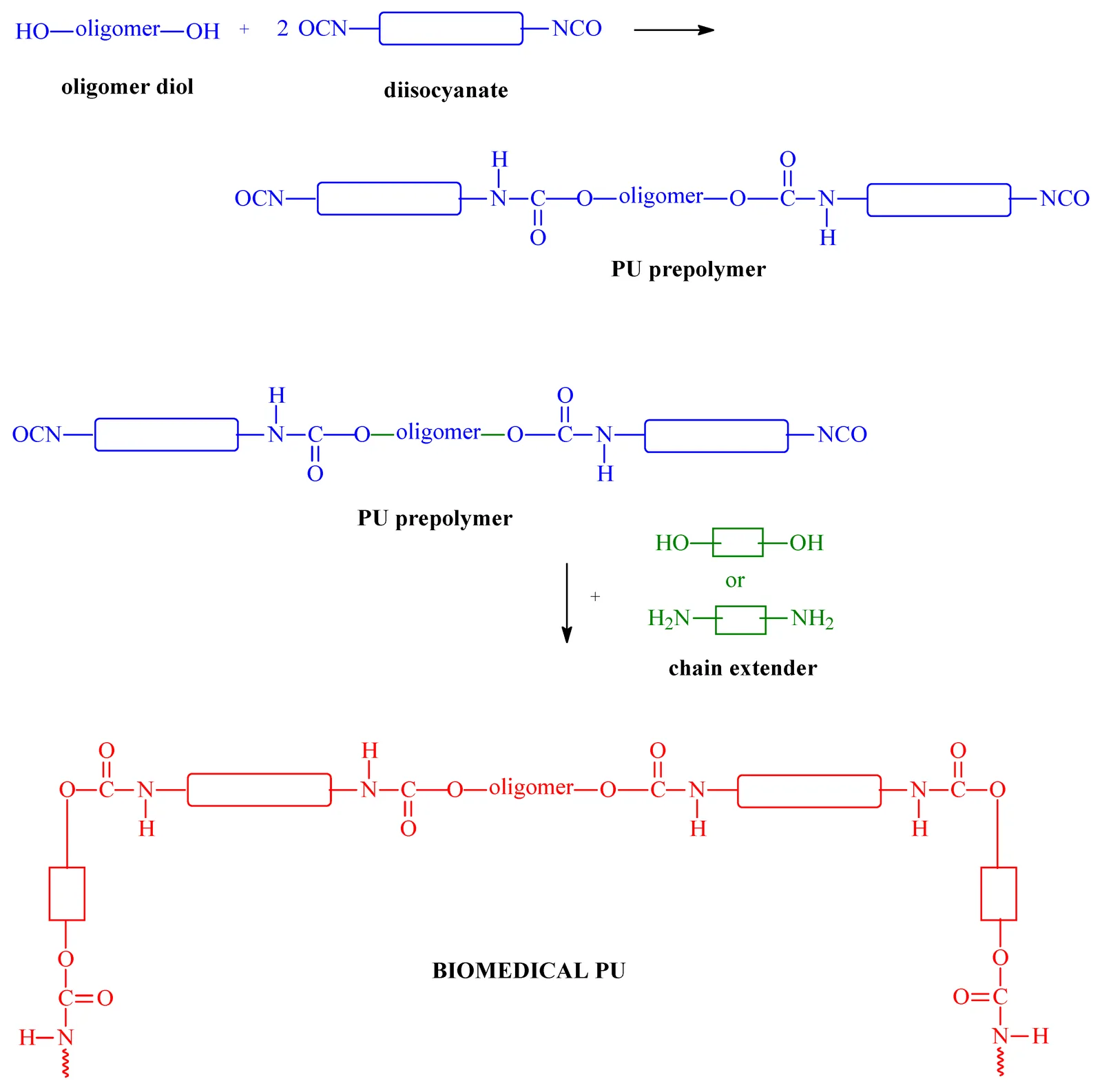
The prepolymer method synthesis of biomedical polyurethanes (applied in tissue engineering).

**Figure 4:**
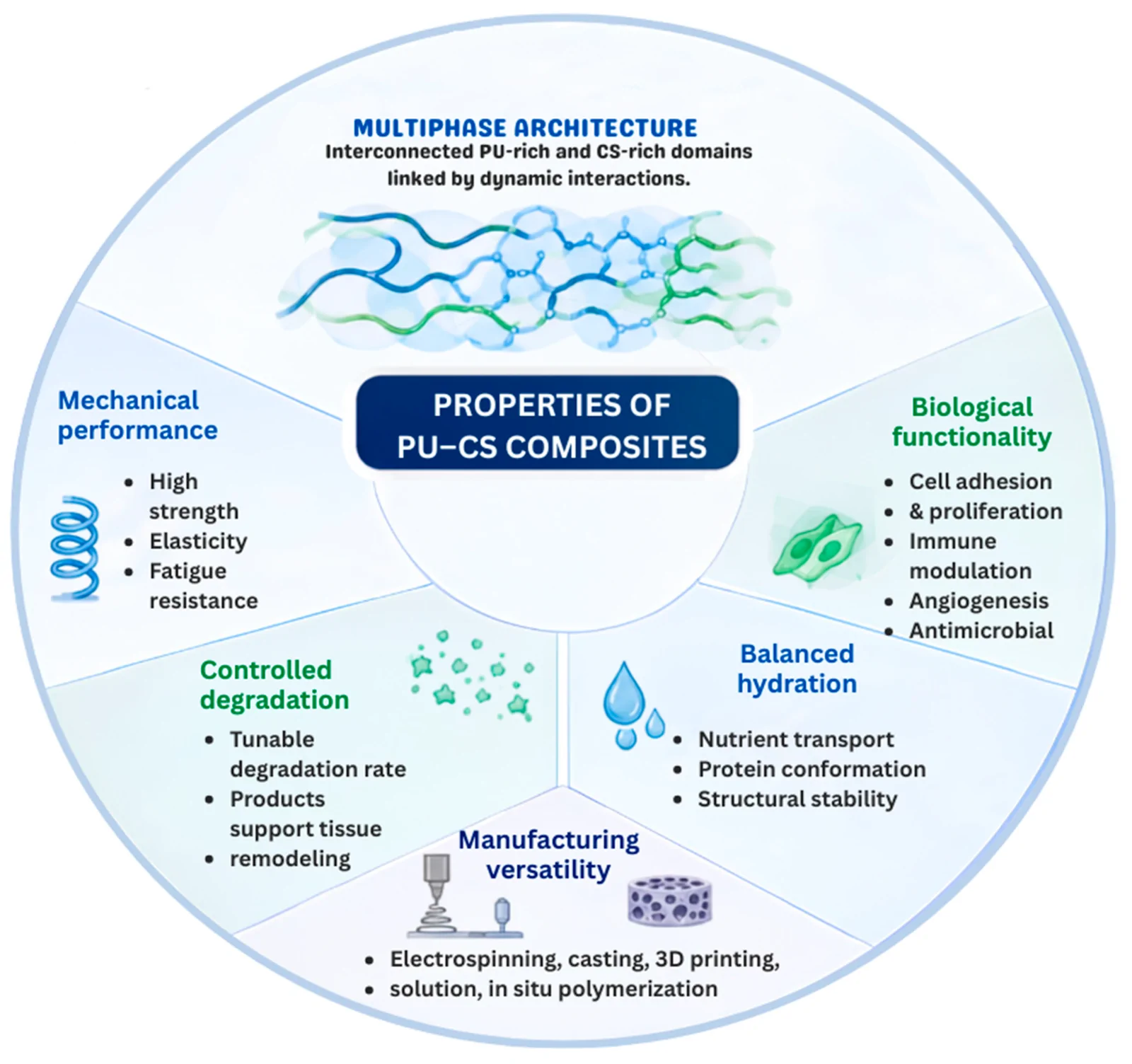
Properties of biomedical polyurethane-chitosan composites. Figure 4 was created by the authors using Canva (version 1.121.0) and Paint (concept, layout, and labels). Certain elements of the figure (hydrogel chains, cells, tissue scaffold, and 3D printer nozzle) were generated using AI and incorporated into the image.

**Figure 5:**
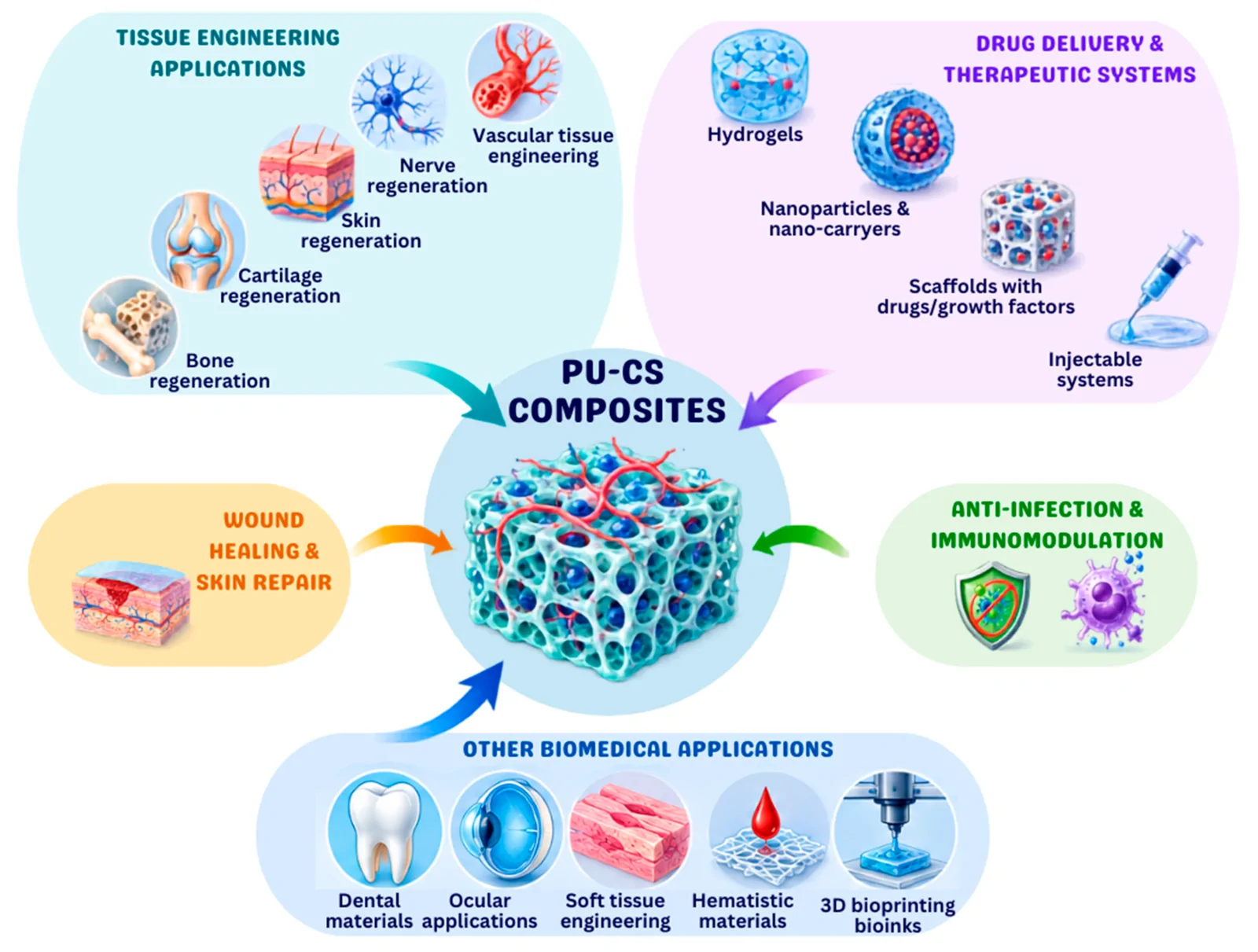
Applications of polyurethane-chitosan composites in tissue engineering and other biomedical applications. Figure 5 was created by the authors using Canva (version 1.121.0) and Paint software (Version 11.2605.81.0) (concept, layout, and labels). Certain elements of the figure (cells, tissues, tissue scaffold, syringe) were generated using AI and incorporated into the image.

**Figure 6:**
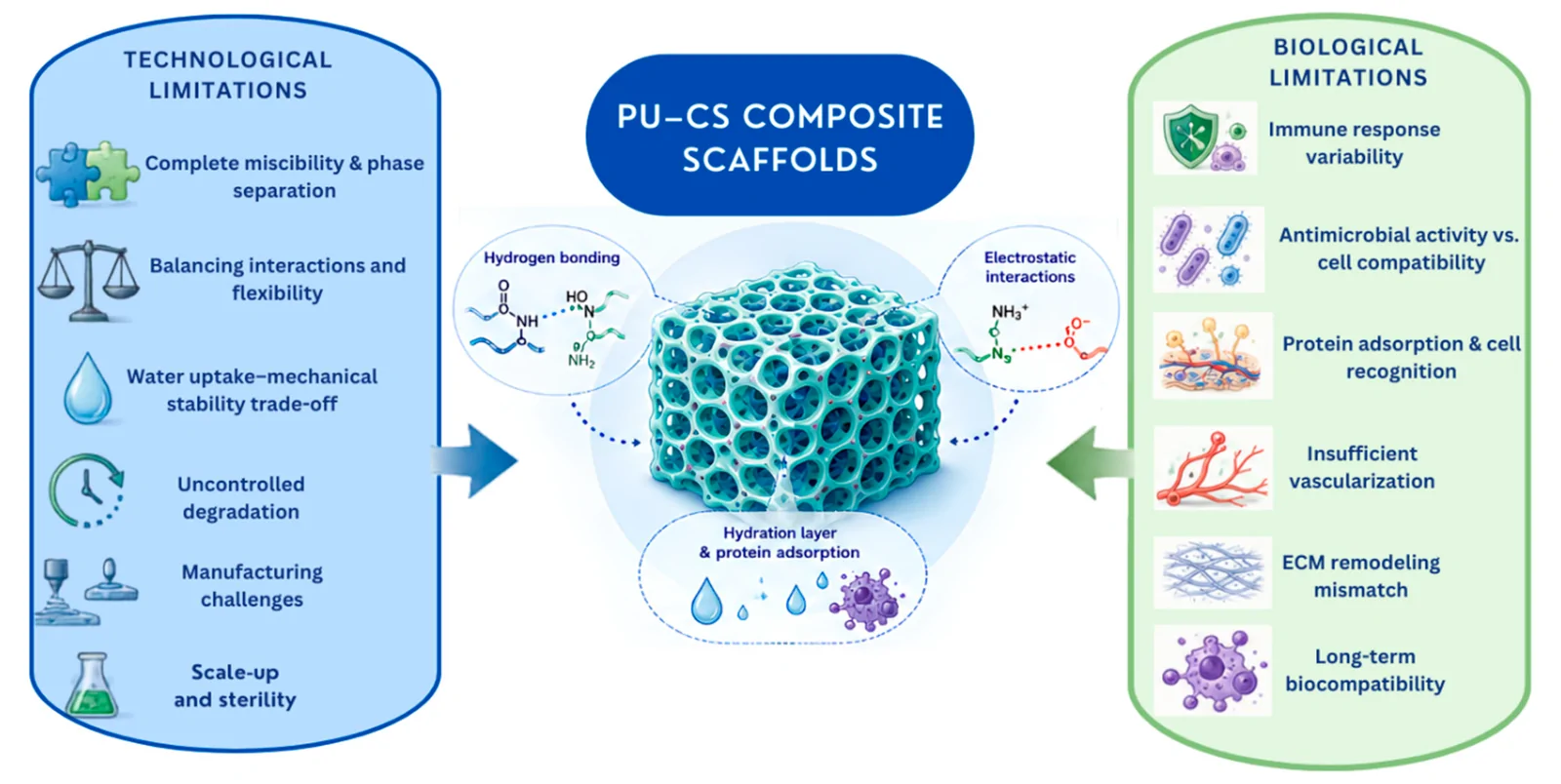
Technological and biological limitations of polyurethane-chitosan composite scaffolds. Figure 6 was created by the authors using the graphics software ChemWin, Canva (version 1.121.0), and Paint (for concept, figure layout, and labels). Certain elements of the figure (cells, tissue scaffold, 3D printer nozzle, scale, and laboratory glassware) were generated using AI and incorporated into the image.

**Table 1:** Examples of biomedical polyurethane-chitosan composites for tissue engineering.

Composite Preparation Method	Polyurethane Characteristics	Chitosan Characteristics	Composite Properties	In Vitro/In Vivo	Advantages	Limitations	Potential Application	Ref.
Aqueous polyelectrolyte assembly/ionic complexation. An anionic WPUs dispersion was prepared first; cationic CS solution was added dropwise to form PU–CS core–shell ionic-complex NPs. The dispersion was cast into films.	Anionic WPUs based on PTMO (M_n_ 1000 g/mol), IPDI, DMPA (internal anionic chain extender), and triethylamine (neutralizing agent); acetone-assisted prepolymer/dispersion method.	CS: DD = 90%; average M_w_ approximately 5000 g/mol. Used as a cationic polyelectrolyte for adsorption/complexation on the PU NPs surface.	Core–shell ionic-complex NPs; Size approximately 60–220 nm. Stable aqueous emulsions and continuous cast films were obtained.	Very low cytotoxicity and supported endothelial-cell adhesion and growth. Prothrombin time was prolonged, indicating improved antithrombogenicity.	Water-based, mild, and readily processable fabrication; combines PU mechanical integrity with CS bioactivity; reduced nonspecific protein adsorption; improved endothelial compatibility and blood compatibility; surface modification without major loss of bulk mechanical performance.	Only in vitro cytocompatibility and haemocompatibility were assessed; Broad particle-size range.	Blood-contacting and cardiovascular tissue-engineering interfaces; endothelialized coatings/films; implantable vascular materials and potentially small-diameter vascular graft surfaces.	[42]
Thermally induced phase separation (TIPS) followed by freeze-drying. PU prepolymer and CS solutions were blended at PU/CS mass ratios of 90/10, 80/20, 70/30, 60/40, and 50/50, frozen at −78 °C, lyophilized for 48 h, and cured at 60 °C under reduced pressure for 48 h.	Prepolymer method. PCL diol (M_n_ 2000 g/mol) and PCL triol (M_n_ 900 g/mol) dissolved in 1,4-dioxane/cyclohexane; HDI added; dibutyltin dilaurate 0.5 wt% used as catalyst. PU/solvent ratio 20% *w*/*v*. Chain extension/crosslinking occurs during curing and through multifunctional polyol/CS reactions.	High-molecular-weight CS; dissolved at 5% *w*/*v* in 0.5 M acetic aci	Highly porous, interconnected 3D scaffolds. CS and PU/CS blends were more porous than PU; morphology depended on blend ratio and TIPS conditions. SEM showed cell spreading and mineral deposits.	Primary rat calvarial osteoblasts adhered and proliferated for up to 28 days. Early attachment depended on PU/CS ratio; CS improved initial adhesion, although early proliferation on some composites was delayed. ALP activity and Ca/P-containing mineral deposition supported osteogenic maturation/mineralization.	Interconnected porosity suitable for tissue ingrowth; tunable composition and morphology; combines PU mechanical adjustability with CS hydrophilicity/bioactivity; supports osteoblast adhesion, growth, and mineralization.	Porosity and pore size were not tightly controlled or comprehensively quantified; early cell proliferation decreased in some formulations; potentially toxic organic solvents/catalysts were used.	Bone tissue-engineering scaffold for osteoblast culture and mineralized tissue formation.	[51]
Covalent surface immobilization of CS on cast PU films. PU surfaces were oxygen-plasma-activated (100 W, 10 min), treated with 50 wt% acrylamide, reacted with 1% glutaraldehyde for 3 h, and then immersed in CS solution (5 or 20 mg/mL in 1% acetic acid) at 4 °C for 24 h; samples were neutralized and washed.	Solvent- and catalyst-free prepolymer/casting route using TDI (80:20 mixture of 2,4-/2,6-TDI) and PEO-PPG. Polyol dried at 80 °C under vacuum for at least 1 h; TDI added under N_2_; stirred 6 h; cast and cured about 15 days at 90 °C under vacuum—no chain extender, catalyst, activator, or solvent.	Low-viscosity CS (<200 mPa·s for 1% solution in acetic acid), DD = 75–85%. Immobilization concentrations: 5 and 20 mg/mL.	The film surface became rougher and more hydrophilic after CS immobilization. The bulk remained a dense, nonporous PU film.	Strong antibacterial and anti-adhesion effects against *S. aureus* and *P. aeruginosa*; colony counts and viable attached bacteria decreased by approximately 10^2^–10^5^ CFU/mL, depending on strain/sample.	Stable covalent CS coating; high antibacterial activity without incorporating antibiotics; improved hydrophilicity and surface roughness; simple modification of preformed medical-grade PU films.	Glutaraldehyde and TDI raise residual toxicity concerns; long curing time; no cytocompatibility, degradation, or in vivo infection model; antibacterial efficacy was stronger against Gram-positive than against Gram-negative bacteria.	Antibacterial surfaces for medical devices, particularly urinary catheters, are potentially useful as infection-resistant tissue-contacting PU membranes.	[44]
Two-step step-growth polymerization. An NCO-terminated PU prepolymer was formed from PCL and TDI (3:1 molar ratio), then chain-extended with varying CS/1,4-butanediol ratios (0/2, 0.5/1.5, 1/1, 1.5/0.5, and 2/0 mol). Cast sheets were vacuum-degassed and cured at 100 °C for 24 h.	PCL-diol (M_n_ approximately 2000 g/mol); 2,4-TDI; BDO and CS as mixed chain extenders. PCL + TDI reacted at 60→100 °C under N_2_ for approximately 1 h; chain extension at 80 °C—no catalyst used.	CS was fragmented with dilute hydrogen peroxide to improve dissolution and then dissolved in DMSO.	Dense elastomeric sheets. Progressive incorporation/distribution of chitosan in the PU matrix; higher CS caused a rougher, more heterogeneous internal morphology and evidence of phase separation/agglomeration.	No biological tests.	CS is chemically incorporated into the PU backbone as a bio-based chain extender; composition is systematically tunable; may improve biofunctionality while retaining elastomeric behavior.	Aromatic TDI and DMSO are less attractive for implant translation; no biological validation, biodegradation, or mechanical dataset; heterogeneity increases at high CS content.	Potential elastomeric biomaterials for soft-tissue-contact devices, sutures, membranes, or scaffolds; tissue-engineering use remains only prospective because biological data were not generated.	[53]
Two-step polymerization of HTPB/HDI PU followed by chain extension with 1,4-BDO and variable CS/CUR blends; cast elastomeric sheets were produced.	HTPB and HDI, with BDO, CS, and CUR as chain extenders, were used.	Commercial CS; CS was used at variable molar fractions together with CUR.	Generally homogeneous surfaces and good dispersion of CS/CUR; composition-dependent microheterogeneity.	Antimicrobial activity was observed; PU obtained from a formulation containing BDO, CS, and CUR (molar ratio: 1:0.75:0.25) exhibited the strongest biological activity among the formulations.	Improved thermal stability at selected CS/CUR ratio; modulus and tensile strength increased with higher CS/CUR content; antimicrobial functionality combined with elastomeric PU.	Biological evidence limited to antimicrobial testing; no cytotoxicity/in vivo data; no porous morphology;	Potential antimicrobial soft-tissue biomaterial, wound-contact elastomer, or coating; direct application as a tissue-engineering scaffold remains unproven.	[55]
Physical blending and drying-induced self-organization/phase separation of aqueous PU-diol dispersion (PUD) and CS, followed by neutralization and ionic crosslinking with 5% STPP. CS: PUD ratios from 10:0 to 3:7 were screened.	Commercial PUD, diluted to 20% *w*/*v* in PBS.	High-molecular-weight CS (M_w_ approximately 710,000 g/mol); DD > 90%. Used as a 4 wt% solution in 2% acetic acid.	Macroporous hydrogel scaffolds generated by drying-induced phase separation. Interconnected pores on top and fracture surfaces. Pore size increased with PUD content up to approximately 16–18 μm; total porosity reached about 90–96%. The wet strength of composite within 7 ± 3 MPa; Highest elongation of 38% for scaffold; Scaffolds showed contact angle of 80° ± 10° (in comparison to CS 62° ± 3°); After 35 days, maximum degradation for composites of 15% was observed and 22% were observed under hydrolytic and enzymatic degradation, respectively;	Biomaterials increased collagen deposition and maturation, as well as neovascularization. Haemocompatibility, protein adsorption, antimicrobial activity, and pH- and enzyme-dependent degradation were also assessed.	Waterborne processing; no toxic covalent crosslinker; high interconnected porosity, swelling, and moist-wound support; composition-dependent mechanics/biodegradation; antibacterial chitosan; strong in vitro and in vivo evidence with comparison to a commercial dressing.	Commercial PUD chemistry is insufficiently defined; STPP crosslink density and long-term fate were not fully addressed; only one animal model and a short healing period; scaffold formability/leaching deteriorated at high PUD fractions;	Skin tissue engineering and full-thickness wound-healing dressing, especially for exudative wounds requiring porous, absorbent, and antimicrobial support.	[62]
PU–CS blend preparation followed by complex coacervation to form the core and ionic gelation of alginate or PU-ALG shell with CaCl_2_. PU: CS and PU: ALG ratio 2.5:1; blends stirred overnight at room temperature. NaOH-induced coacervation and sonication produced core particles; alginate shells were crosslinked with 10 mg/mL CaCl_2_.	Hydrophilic PU synthesized from recycled PET-derived BHET, HDI, and PEG 600. PET glycolysis: ethylene glycol + zinc acetate at 190 °C under N_2_; BHET purified. PU prepolymer: HDI 0.02 mol + BHET 0.013 mol at 130 °C for at least 1 h; PEG 600 (0.008 mol) added, and reaction continued at 180 °C to yield hydroxyl-terminated PU. Prepolymer method.	CS (M_w_ = 230 kDa); DD = 86%. Dissolved in 1% acetic acid.	Core–shell NPs with chitosan or PU–CS cores and ALG or PU–ALG shells. PU primary particles were approximately 5–15 nm; final insulin-loaded core–shell particles were nanoscale, composition-dependent, and more irregular when PU was present in both core and shell. Core–shell structures and pH-dependent swelling.	High insulin encapsulation (up to approximately 82.3% in optimized formulations), low release at pH 1.2, and sustained intestinal-pH release. Strong mucoadhesion. In alloxan-diabetic mice, oral formulations reduced blood glucose and prolonged hypoglycemia; relative insulin bioavailability was 9.04–11.6%, higher than basic CS/ALG particles. Acute toxicity/biochemistry indicated acceptable short-term safety.	Valorizes waste PET; protects protein drug in gastric conditions; improves encapsulation, controlled release, mucoadhesion, and oral bioavailability; combines biodegradable polysaccharides with hydrophilic PU; in vivo pharmacological validation.	High-temperature PU synthesis and zinc-acetate purification; limited long-term toxicity/immunogenicity data;	Oral protein-drug delivery and gastrointestinal mucosal biomaterials;	[59]
One-pot casting/crosslinking synthesis of castor-oil-based PU with dispersed CS. Castor oil was used directly or first transesterified with pentaerythritol; polyol/CS suspensions were reacted with IPDI at NCO/OH = 1. CS contents of 2.5, 5, and 7.5 wt% were evaluated.	Original castor oil (hydroxyl index 163 mg KOH/g) or modified castor-oil polyols. Modification: 120 °C/10 min drying, then 180 °C for 2 h with pentaerythritol and 0.05 wt% PbO catalyst. PU: IPDI, NCO/OH = 1, no separate low-molecular chain extender. The PU network was crosslinked due to multifunctional polyols. Materials were thermoset/amorphous networks.	Low-molecular-weight CS, DD = 75–85%; Added at 2.5, 5, or 7.5 wt%.	Dense crosslinked PU. Increasing chitosan agglomeration and phase separation with rising CS concentration, particularly at the highest loading, altered the uniformity of the polyol-modified matrix. Hardness (Shore A): 45–84.	In vitro degradation at 37 and 105 °C, PBS swelling/contact angle, and direct/indirect L929 fibroblast viability were measured. Cell viability remained >70% for solid polymers and biodegradation products, meeting a non-cytotoxicity criterion.	Renewable castor-oil polyols; tunable crosslink density/hardness; CS incorporation confirmed chemically; non-cytotoxic solid materials and degradation products; simple solvent-free-type network fabrication.	Hydrophobic surfaces (contact angle > 65°), limited swelling and biodegradation response; CS agglomeration/phase separation at higher loading; PbO catalyst is a major translational toxicology concern; no porous scaffold;	General non-load-bearing biomedical elastomers, coatings, or implant components; potential soft-tissue biomaterials.	[46]
Step-growth prepolymer polymerization with in situ incorporation of 1 wt% montmorillonite (MMT). TDI/HTPB prepolymer was chain-extended with variable CS/BDO molar ratios (0/2, 0.5/1.5, 1/1, 1.5/0.5, 2/0). CS was dissolved in DMSO; sheets were cast and cured for 24 h at 100 °C.	Prepolymer route. Polyol: HTPB. Diisocyanate: TDI (3 mol TDI: 1 mol HTPB). Chain extenders: BDO and/or CS. MMT: 1%. Reaction: 60→90 °C under N_2_; NCO-prepolymer formation ~1 h; chain extension 30–40 min at 90 °C. CS increased thermal stability and altered phase organization.	CS, DD = 80%. Dried at 90 °C under vacuum for 24 h and dissolved/treated in DMSO before chain extension.	Dense cast bio-nanocomposite sheets. Incorporation of CS and generally good MMT dispersion; higher CS changed surface texture and domain organization.	Antibacterial disc diffusion testing showed activity against selected bacterial strains, with activity increasing with CS-containing formulations.	Chemical incorporation of CS as a chain extender improved thermal stability, antibacterial activity, MMT reinforcement, and composition-dependent property tuning.	No cytotoxicity, hemocompatibility, biodegradation, cell-adhesion, or in vivo evaluation; aromatic TDI and HTPB may raise biodegradation/biocompatibility concerns; catalyst; cast films lack interconnected porosity.	Potential antimicrobial biomedical films, wound-contact materials or device coatings;	[48]
Surface biofunctionalization of castor-oil PU films: low-pressure N_2_ plasma activation → acrylic-acid graft polymerization → EDC/NHS activation of surface COOH groups → covalent immobilization of CS (or collagen).	Prepolymer/casting route. Polyol: castor oil. Isocyanate: commercial HDI trimer. Castor oil reacted with HDI at 80 °C under N_2_ for 2 h after 30 min addition, forming a viscous prepolymer; 600 µm films were cast and cured for 24 h at 80 °C. Films were extracted in 70% ethanol, then vacuum-dried.	Medium-molecular-weight commercial CS. Immobilized from aqueous acidic solution after EDC/NHS activation.	Flexible, transparent films. Plasma/PAA/biomolecule treatment increased nanoscale roughness and changed topography. CS-immobilized surfaces exhibited bump- and strand-like features and increased wettability compared with untreated PU.	In vitro L929 fibroblast assays: CS- and collagen-immobilized films improved cell adhesion and proliferation compared with untreated PU; CS-modified PU exhibited the strongest antibacterial activity.	Surface-selective modification preserves bulk PU mechanics; enhanced hydrophilicity, fibroblast response, and antibacterial function; covalent immobilization improves coating stability; transparent/flexible wound-contact format.	Multistep processing (plasma, PAA grafting, EDC/NHS chemistry); no degradation or in vivo wound-healing test; possible residual coupling reagents require control.	Wound dressing and skin-contact biomaterial; potential skin-tissue-engineering membrane or antibacterial coating.	[43]
In situ fabrication of CS-promoted PCL-based shape-memory PU films. H_2_O_2_-treated/freeze-dried CS nanoflakes were swollen in DMF, mixed with PCL-diol, reacted with HDI to form an NCO-terminated prepolymer, chain-extended with BDO, diluted with chloroform, cast, and vacuum-cured.	Prepolymer method. PCL-diol (M_n_ 2000 g/mol); HDI; BDO chain extender; DBTDL catalyst. CS contents 5, 7.5, 10, and 20 wt% relative to PCL. Reaction at 80 °C for 2 h under inert atmosphere; final NCO:OH = 1:1.1; films vacuum-dried 24 h at 80 °C. Semicrystalline PCL switching phase; stress-induced crystallites generated during stretching; crystallinity decreased/changed with CS but enabled thermal shape recovery.	Shrimp-shell CS, DD ≥ 75%. Pretreatment: 30% H_2_O_2_, 40 °C, 24 h; precipitation in acetone, washing, freeze-drying, and DMF swelling. Produced nano- to microscale flakes dispersed in the PU matrix.	Continuous films with CS nanoflakes homogeneously embedded; nanoflakes acted as multifunctional crosslinking nodes. At excessive CS loading, aggregation/heterogeneity became more likely. No interconnected porous scaffold. Ultimate tensile strength of composites was 12 MPa (for PU was found to be 5.2 MPa); The elongation at break for PU was found to be 102%, which increases to 598% after the addition of 10% CS in the PU-CS biocomposite.	Physicochemical testing showed >500% elongation at break, ~130% increase in ultimate tensile strength, ~100% shape recovery, and T_max_ ~360 °C. Biodegradability was discussed/assessed materially, but biological safety was not established.	Outstanding extensibility and toughness; near-complete thermally triggered shape recovery after large deformation; biodegradable PCL basis; aliphatic diisocyanate; CS reinforcement and antibacterial potential.	No cytocompatibility/in vivo validation; film architecture not inherently porous; activation temperature and long-term fatigue under physiological conditions require study; high CS may impair uniformity.	Potential deployable/self-fitting biomedical devices, minimally invasive implants, soft-tissue supports, and shape-memory scaffolds; application remains prospective.	[47]
Two-step synthesis and chemical foaming of PEG-based PU using CS of different molecular weights as chain extenders. IPDI/PEG prepolymer was mixed with CA, CS3K, CS30K, or CS300K solution and foamed using catalyst/surfactant components.	Prepolymer method. Soft segment: PEG (Mn 2000 g/mol). Diisocyanate: IPDI. CS incorporated at 5 wt% as a chain extender. Catalysts: SnOct_2_ and triethylenediamine (DABCO); silicone L-580 surfactant; water/foaming formulation. CS grafting degree: high for CA and CS3K (reported 78.9% and 98.3%). PU-CS3K retained a diffuse crystalline peak similar to PU; CA disrupted ordering; CS30K/CS300K caused CS/soft-segment phase separation.	CA; chito-oligosaccharide (Mn 3000; 30,000 and 300,000 g/mol). All CS grades DD > 90%.	All formulations formed 3D cellular foams. PU and PU-CA were relatively uniform; PU-CS3K showed dense, small interconnected/through pores; CS30K and CS300K produced irregular pores, thicker walls, and phase-separated/disordered bubble structure. Tensile strength (KPa) (dry/wet): 30–33/5–16; Elongation at break (%) (dry/wet): 58–64/26–39; Young’s modulus (MPa) (dry/wet): 47–57/18–41.	No cell or animal study. BSA adsorption increased after CS incorporation. Five-week SBF degradation: PU ~60% mass loss; CS-containing foams reached approximately 70–98% depending on CS grade (highest for high-M_w_ CS). Mechanical, wetting, absorption, and thermal tests demonstrated strong molecular-weight dependence.	Interconnected porous architecture; tunable mechanics, degradation, and protein adsorption; high-M_w_ CS improves dry/wet strength; low-Mw CS gives higher grafting and more uniform pores; DD and Mw were explicitly controlled.	No cytotoxicity or in vivo data; high-M_w_ CS causes phase separation and irregular pores; low-Mw CS may reduce tensile strength and initially slow hydrolysis through high crosslinking; hydrophobicity/protein adsorption increase with CS Mw; catalyst residues not biologically assessed.	Candidate absorbent wound scaffold and general porous biomedical matrix; potential soft-tissue or skin scaffold, but tissue-specific cell validation is required.	[52]
Synthesis of CS-containing biodegradable PU by prepolymer method followed by solution electrospinning. Three PUs (PU-DMPA, PU-DMPA/CS, and PU-CS) were prepared; fiber mats were electrospun from DMF/THF and compared with solvent-cast films.	Prepolymer method. PCL-diol (M_n_ 2000 g/mol) dried 24 h/90 °C; IPDI added at 60→90 °C and reacted 4 h. Chain extenders: DMPA and/or depolymerized CS. DMPA neutralized with TEA; CS reacted in DMSO; NaHSO_3_ used to terminate residual NCO; polymer precipitated, washed, and dried. Fiber mats had a higher crystalline degree than corresponding films; CS increased modulus, and PU-CS gave improved recovery/resilience.	CS: M_w_ 500 kDa, DD = 75.5%. Oxidatively depolymerized with NaNO_2_ to M_v_ 26 kDa and dissolved in DMSO before use as a chain extender.	Random nonwoven electrospun fibrous mats with interconnected pores and fibre diameter around 1 µm. Fibres exposed ester and urethane groups in similar proportions. Films were dense. CS-containing fibres showed suitable ECM-like architecture. Young’s modulus increased with CS in composites content and was higher for films (2.5 MPa to 6.5 MPa) than for the corresponding fibre mats (0.8 MPa to 3.2 MPa).	Human dermal fibroblast testing: no sample was cytotoxic; cells adhered better to fibres than films; proliferation occurred only on CS-containing PUs. Both films and mats underwent hydrolytic biodegradation. No in vivo study.	Chemically integrated CS; biodegradable PCL soft segment; fibrous ECM-like morphology; CS-dependent stiffness, wettability and resilience; favourable fibroblast adhesion/proliferation; aliphatic IPDI.	Organic solvents (DMF/THF, DMSO/NMP) and extensive purification; fibre diameter ~1 µm; no in vivo or long-term inflammatory assessment; mechanical properties lower for mats than films; biodegradation-rate kinetics not established.	Soft-tissue-engineering scaffold, particularly dermal/skin, vascular, or nerve-support matrices.	[63]
Coaxial electrospinning of drug/MOF-loaded core–shell fibres. Core: CMCS /PEO (8:2) containing DOX- and FA-loaded UiO-66 nanoparticles; shell: commercial PU in DMF. Core flow 0.5 mL/h; shell flow 0.3, 0.5, or 0.8 mL/h.	Commercial PU. Shell solution: 10 wt% PU in DMF. PU served as a hydrophobic diffusion-controlling shell.	CMCS, Mw 100–250 kDa, N-deacetylation ≥95%. Core solution: 5 wt% CMCS with 5 wt% PEO (PEO Mw 900 kDa), CMCS /PEO 8:2.	Continuous core–shell nanofibres containing UiO-66 nanoparticles; fibrous core–shell morphology. Shell-flow rate changed diameter and barrier thickness. MOF/drug particles were distributed within the hydrophilic core protected by the PU shell.	In vitro MCF-7 breast-cancer assays (MTT, DAPI, flow cytometry): DOX/FA core–shell fibres caused up to 82 ± 0.7%, 83 ± 0.5% and 87 ± 0.5% cell death for increasing shell-flow formulations after 168–240 h. Controlled release exhibited diffusion behavior that depended on the architecture. No normal-cell, tissue-regeneration, or in vivo study.	High loading capacity and sustained/local release; PU shell suppresses burst release; simultaneous DOX and FA delivery; high-surface-area fibrous platform; tunable release by shell flow.	Primarily an anticancer drug-delivery system rather; commercial PU chemistry undisclosed; no in vivo efficacy, normal-cell safety, or biodegradation study; UiO-66/zirconium fate and residual DMF require evaluation.	Local anticancer implant or post-resection tumour-bed dressing; potential theranostic fibrous matrix.	[58]
Step-growth prepolymer synthesis of starch/CS-modified PU sheets. IPDI/HTPB NCO-terminated prepolymer was chain-extended with variable mixtures of CS, acid-hydrolysed starch and/or BDO dissolved in DMSO; resin was cast on Teflon and oven-cured.	Prepolymer method. Polyol: HTPB. Diisocyanate: IPDI, 3:1 molar ratio to HTPB. Chain extenders: BDO, CS and acid-hydrolysed corn starch. Prepolymer prepared at 100 °C under argon for ~1 h. An equal starch/CS combination gave the highest thermal stability among blends.	CS was dried and dissolved in DMSO for chain extension.	Dense cast sheets/films. Composition-dependent surface morphology and confirmed incorporation/dispersion of polysaccharide components; no designed interconnected macroporosity.	No in vitro cell, antibacterial, hemocompatibility, biodegradation, or in vivo study.	Uses two renewable polysaccharides; chemical incorporation into PU; improved thermal stability at balanced starch/CS composition; simple composition tuning.	HTPB may be poorly biodegradable; no porosity, cell response, biodegradation kinetics tests, or animal evidence.	Prospective biodegradable biomedical films, wound/tissue-contact sheets, or general biomaterials.	[54]
Gamma-radiation-induced grafting of CS onto P(3HB), followed by step-growth PU-urea synthesis using the graft copolymer as chain extender; silica NPs were added, and salt-leaching generated a 3D porous scaffold.	Poly(saccharide-ester-urethane/urea) route. P(3HB) (M_w_ 60,000 g/mol) was grafted with CS. BDI and PEG were used in the proposed PU synthesis, with P(3HB)-g-CS serving as a multifunctional chain extender. Silica NPs from vermicomposting acted as reinforcement/bioactive filler.	CS (M_w_ 50–190 kDa; DD = 75–85%). CS was grafted to P(3HB) by gamma-radiation-induced polymerization before PU formation.	3D salt-leached composite foams with high porosity and interconnectivity. Silica NPs reinforced the network; swelling and compression depended on solvent/preparation conditions.	PU-urea composites were non-cytotoxic in the reported assays and supported 3D culture of pancreatic islet cells with suitable viability.	Combines biodegradability of P(3HB)/CS with a mechanically reinforced PU network; high interconnected porosity; supports sensitive pancreatic islet cells; incorporates waste-derived silica; non-cytotoxic in vitro.	Gamma-grafting scalability and reproducibility need evaluation; silica source variability; residual porogen/solvent control.	3D pancreatic islet-cell culture and potential islet transplantation scaffold; broader soft-tissue/organotypic culture platform.	[45]
Two-stage waterborne PU-CS hydrogel synthesis. PLGA-diol was first prepared by ring-opening/polycondensation of lactide/glycolide with ethylene glycol. HDI/PLGA-diol/DMPA prepolymer was synthesized in DMSO, neutralized with TEA, and then an unmodified CS solution was added dropwise; membranes were cast and thermally crosslinked.	Prepolymer method. PLGA-diol (M_n_ ~2000 g/mol), LA:GA 50:50; HDI; DMPA internal emulsifier (7 wt% relative to polyester diol); TEA neutralizer; SnOct_2_ as catalyst. NCO(HDI):OH(PLGA):OH(DMPA) = 2:1:1; prepolymer 70 °C, 2 h under N_2_. CS reacted during casting at 60 °C/24 h through urethane/urea bonds. Amorphous/crosslinked hydrogel.	CS: M_v_ 1500 kDa; DD = 75%. Dissolved at 0.01 g/mL in 1% acetic acid at 40 °C using ultrasound.	Crosslinked hydrogel membranes; composition-dependent swelling (~1200–2000% range reported) and phase behaviour. HPUC with the highest prepolymer content showed phase separation; the network remained insoluble and underwent hydrolytic degradation over weeks.	Low cytotoxicity toward bone-marrow-derived cells. In diabetic rats, HPUC, when used with the bone marrow mononuclear fraction, improved ulcer wound healing compared with controls, as evidenced by histology. The material acted as a membrane/vehicle for cell therapy.	Waterborne/crosslinked hydrogel; high swelling; tunable PLGA-driven hydrolytic degradation; chemically integrated CS; supports cell transplantation; direct diabetic-wound in vivo evidence.	High-M_w_ CS is difficult to process; phase separation at high PU fraction; combination therapy makes it difficult to isolate scaffold-only contribution; limited long-term biodegradation/immunogenicity and mechanical data; residual catalyst/solvent must be controlled.	Diabetic ulcer treatment, wound dressing, and cell-delivery membrane for skin tissue engineering.	[41]
Physical blending and electrospinning of commercial thermoplastic PU with CS and MWCNTs. Random or aligned PU/CS/CNT fibres were produced; CNTs were either blended into the spinning solution or electrosprayed onto fibres. Selected scaffolds were glutaraldehyde-treated/crosslinked.	Commercial medical-grade thermoplastic PU Tecoflex SG-80A; PU solutions 5–7 wt% in 90% acetic acid; 6 wt% selected. No PU synthesis catalyst or chain extender used.	Low-molecular-weight CS (M_w_ ~100 kDa). CS solution 2 wt% in 90% acetic acid. PU:CS ratios 1:3, 1:1 and 3:1.	Random and aligned nanofibrous mats. Optimal 6% PU fibres ~229 ± 30 nm; increasing CS reduced mean diameter (approximately 169 ± 25 to 103 ± 18 nm). CNT-containing fibres remained nanoscale but could show CNT agglomeration when blended; electrospraying improved surface distribution. Alignment obtained at 2500 rpm. The ultimate tensile strength of PU nanofibers was 5.66 MPa which was increased to 9.46–12.3 MPa in CS containing samples based on the ratio of PU/CS. The PU/CS/CNT nanofibers exhibited tensile strength of 14.24 and 22.3 MPa in PU/CS/CNT [3:1] and [1:1] samples, respectively.	In vitro H9C2 rat cardiac myoblasts and HUVEC assays confirmed biocompatibility and proliferation. Conductive CNT-containing scaffolds supported cell growth; electrosprayed CNT scaffold showed particularly favourable proliferation. PBS degradation/mass retention assessed up to 2 months. No in vivo myocardial study.	ECM-like aligned architecture; electrical conductivity; improved tensile properties (CS increased strength; CNT enhanced functionality); tunable fibre orientation; supports cardiac and endothelial cells; potential directional guidance.	No in vivo cardiac function, contractility, or electrophysiological coupling test; CNT agglomeration/release and chronic safety are concerns; acetic acid and glutaraldehyde processing require residue control.	Cardiac tissue-engineering patch for regeneration of infarcted myocardium; aligned conductive scaffold for cardiomyocyte organization.	[64]
PCL-PU-urea was synthesized by a two-step prepolymer route and then physically blended with CS. A 6% (*w*/*v*) PCL-PU-urea solution in DMF was mixed 1:1 with 3% (*w*/*v*) CS in 2% acetic acid, homogenized for 1 h, and freeze-dried. The scaffold was purified in ethanol and oven-dried.	PCL-diol (M_w_ 2000 g/mol) was reacted with HDI and putrescine as diamine chain extender at a 2:1:1 molar ratio. SnOct_2_ (0.5 wt%) was the catalyst. Prepolymerization: 75 °C, 3 h under inert gas; chain extension: 25 °C, 18 h. PCL-PU-urea was semicrystalline, and its interaction with CS increased thermal stability and compressive strength.	Medium-molecular-weight CS; DD = 75–85%. CS was used at 1.5% in the final scaffold and dissolved in 2% acetic acid.	Freeze-dried, dense, and relatively homogeneous composite scaffold. CS increased surface roughness and hydrophilicity. Hydrogen-bond interactions between PCL-PU-urea and CS were indicated. The composite showed mixed amorphous/semicrystalline character. The compressive stress of the PU and PU/CS was recorded at 7.79 and 15.82 MPa where the ultimate strain of them was 58.28 and 88.61, respectively.	Non-cytotoxic toward human bone-marrow mesenchymal stem cells. Supported adhesion and proliferation. Increased ALP activity, calcium deposition, and expression of Runx2 and osteocalcin. Compressive strength reached 15.82 MPa. No in vivo study.	Good compressive strength; enhanced wettability and roughness; biocompatibility; promotion of osteogenic differentiation and mineralization; biodegradable biomaterials.	No animal implantation tests; long-term biodegradation study; residual-solvent removal is critical; pore architecture was less open than in highly porous bone scaffolds.	Bone tissue engineering and regeneration; implantable scaffold supporting osteogenic differentiation of hBMSCs.	[40]
Modified CS was blended with thermoplastic PU and electrospun. Linezolid was incorporated at different doses into the spinning formulation. Homogeneous nanofibrous wound dressings were obtained and applied to full-thickness wounds in streptozotocin-induced diabetic rats.	Commercial thermoplastic PU was used as the synthetic phase. PU was processed by solution electrospinning.	Modified CS was used. It was blended with thermoplastic PU and used as the hydrophilic/biological component.	Uniform, homogeneous, small-diameter electrospun filaments. Linezolid incorporation did not adversely alter the nanofiber morphology.	In vivo diabetic-rat study: dressings containing linezolid at 1, 3, or 5 mg/kg promoted wound healing and controlled microbial growth compared with drug-free PU/modified-CS fibers. The material supported epidermal cell and growth factor infiltration.	Combines ECM-like nanofibrous architecture, CS bioactivity, and local antibiotic delivery; improved infection control; potential replacement for conventional gauze; direct evidence from a diabetic wound model.	No long-term biosafety or systemic linezolid exposure analysis; antibiotic resistance and dose-translation issues; animal model only.	Antimicrobial wound dressing for acute and chronic diabetic wounds; local antibiotic delivery and skin regeneration.	[39]
CS NPs were fabricated by ionic gelation with TPP, blended with commercial PU and elastin (0.5, 1.0, or 1.5%), and electrospun from DMF/Tris-based solutions. Electrospinning: 12 kV, 0.5 mL/h, 21-G needle, 15 cm distance, and 8 h collection.	Commercial Tecoflex EG-80A PU, 8% (*w*/*v*) in DMF.	Commercial CS; NPs 90–130 nm were obtained in 1% acetic acid by ionic gelation with TPP at a CS:TPP ratio of 5:2, followed by centrifugation and drying.	Continuous electrospun nanofibers with enlarged fiber diameter, pore size, and porosity after CS/elastin addition. Porous architecture was favorable for tissue integration.	Improved wettability by up to 39.6% and faster degradation. APTT increased by 26.8%; minimum hemolysis 2.6 ± 0.1%. CS and elastin were biodegraded in a controlled manner.	Antithrombogenic and hemocompatible surface; tunable porosity and degradation; natural polymers compensate for PU hydrophobicity; simple electrospinning process.	Potential rapid loss of elastin/CS during degradation; mechanical compliance and suture retention require further validation.	Biodegradable vascular membranes, vascular graft interfaces, and cardiovascular tissue replacement.	[65]
A biodegradable waterborne dialdehyde polyurethane (DP) was synthesized, combined in situ with TEMPO-oxidized CNF to form a nanoparticulate crosslinker, and directly blended with aqueous CMCS. Hydrogels formed in about 33 min; films were cast, dried, and tension-annealed at 60 °C under 0–250% pre-strain.	PCL-diol (M_n_ = 2000 g/mol)/IPDI/DMPA WPUs. PCL:IPDI:DMPA = 1:2.7:1; SnOct_2_ catalyst; 75 °C for 3 h, then DMPA/MEK for 1 h, TEA neutralization at 50 °C for 30 min, EDA chain extension in water for 1 h, and glyoxal end-capping for 30 min. DP was nanoparticulate and aldehyde-functional.	CMCS, M_w_ = 20 kDa. Amino groups reacted with aldehyde end groups through dynamic imine-type crosslinking.	Injectable, shear-thinning hydrogel with porous freeze-dried morphology; cast films approximately 50 ± 5 μm thick. Tension annealing aligned the microstructure and produced marked mechanical anisotropy. The Young’s modulus of composites at 25 °C was 67–203 MPa and that at 60 °C was 32–57 MPa; The elongation at the break of composites was 8–15 at 25 °C, and was 16–335% at 60 °C.	Composite films achieved approximately 420% elongation at 60 °C and an anisotropy ratio of about 4.9 after 200% pre-strain. Neural stem cells oriented along the anisotropic direction. Cytocompatibility was demonstrated.	Waterborne processing; injectability and self-healing behavior; high stretchability; controllable anisotropy; cell-guiding ability; biodegradable PCL/CMCS/CNF composition.	Complex multicomponent synthesis; glyoxal/imine stability and residual-reactant safety require further study; no long-term biodegradation or neural-regeneration animal model; mechanical behavior depends on thermal annealing and pre-strain.	Anisotropic biomimetic scaffolds, particularly neural tissue engineering and other aligned soft tissues.	[66]
Commercial PU containing 1–5 wt% Mg(OH)_2_ nanoparticles was electrospun from THF/DMF. The fibers were subsequently hydrothermally coated with CS and silver NPs for 1 h at 80 °C.	Commercial thermoplastic PU Tecoflex EG-80A, reported M_w_ approximately 110,000 g/mol. Electrospinning solution: 11 wt% PU in THF/DMF 90:10; 13.5 kV, 15 cm, 184 rpm collector.	CS, DD = 90%. CS was used as a hydrothermal surface coating and as a reducing/stabilizing environment for Ag deposition.	Fibrous scaffolds with diameters ranging from 0.79 ± 0.31 to 2.5 ± 0.9 μm. Mg(OH)_2_ reduced fiber diameter, whereas the CS/Ag hydrothermal coating increased it and produced a rough, highly hydrophilic surface.	Contact angle decreased from 105.53 ± 0.8° for PU to 9.3 ± 0.8° after Mg(OH)_2_/CS/Ag modification. SBF incubation demonstrated apatite formation. 3T3-L1 cells adhered, proliferated, and reached confluence; MTT showed cytocompatibility. Antibacterial functionality was attributed to Ag.	Excellent wettability; biomineralization; combined osteoinductive Mg(OH)_2_, bioactive CS, and antibacterial Ag; ECM-like fibrous structure; commercial PU provides mechanical support.	No animal bone-defect study; potential silver-ion cytotoxicity and long-term release were not fully resolved; commercial PU is only slowly degradable; coating uniformity and reproducibility may be difficult at scale.	Bone tissue engineering, osteoconductive and antibacterial bone-regeneration scaffold.	[67]
Commercial PU, CS, and collagen solutions were blended with saffron-extract-synthesized Au NPs and electrospun into random or aligned mats. PU/CS and PU/collagen/CS blends were prepared at 75:25; electrospinning used 20 kV, 1 mL/h, 10 cm distance, and collector speeds of 150 or 1500 rpm.	Biomedical-grade commercial thermoplastic PU Tecoflex SG-80A. PU was used at 7 wt% in glacial acetic acid.	Low-molecular-weight CS, DD = 91.2%. CS was used at 2 wt% in 90% acetic acid and blended with PU, with or without collagen.	Random and highly aligned nanofibrous mats. AuNPs were about 13 nm with a zeta potential of around −37 mV. Addition of CS, collagen, and AuNPs increased hydrophilicity and surface nanoroughness while retaining continuous fibers. Incorporation of gold nanoparticles into the polymeric nanofibers could result in the less contact angles of 58 ± 2° and 54 ± 2° for random and aligned PU/CS/AuNPs, respectively. Random and aligned PU/Col/CS nanofibers showed water contact angles of 63 ± 2° and 59 ± 2°, respectively.	In vitro degradation was followed for 60 days in PBS. Tensile properties were considered suitable for cardiac patches. HUVEC alamar-blue assays showed appropriate cell survival.	ECM-like aligned structure; hydrophilicity and biological cues from CS/collagen; AuNPs may enhance nanoroughness and conductivity; acceptable tensile behavior and endothelial cytocompatibility.	No direct electrophysiological or cardiomyocyte maturation evidence; no in vivo infarction model; AuNP long-term retention and biosafety need study; collagen source variability.	Cardiac tissue regeneration and electrospun cardiac patches, especially aligned scaffolds for myocardial repair.	[68]
Star-shaped and linear PCL were synthesized by ring-opening polymerization, modified with HDI to introduce urethane linkages, mixed with CS solution, and frozen/freeze-dried to create physically crosslinked cryogels. Networks were formed mainly by hydrogen bonding rather than covalent PU-CS crosslinking.	PCL/pentaerythritol 100:1, SnOct_2_ catalyst, 120 °C for 24 h under N_2_. Linear PCL used 1-dodecanol initiator under analogous conditions. PCL/HDI molar ratio 1:3; LPCL was likewise urethane-modified in DMSO to form Star-shaped PCL-PU and linear PCL-PU. Linear PCL-PU exhibited higher crystallinity than star-shaped PCL-PU.	CS, M_w_ = 45,000 g/mol, DD = 75%. Amino and hydroxyl groups served as hydrogen-bond donors/acceptors in the cryogel network.	Macroporous, shape-recoverable cryogels. Star-shaped cryogels had larger pores and higher swelling because multiarm PCL-PU restricted chain alignment; linear cryogels had smaller pores, better alignment, and higher crystallinity. The compressive strength was 103 kPa.	All cryogels were non-cytotoxic to MC3T3-E1 preosteoblasts and promoted cell adhesion. Linear cryogels showed higher compressive strength; star-shaped cryogels showed higher swelling and larger pores.	Simple physical crosslinking; no additional toxic chemical crosslinker; tunable pore size, swelling, and compression through PCL-PU structure; rapid shape recovery; cytocompatibility.	Physical hydrogen-bond network may weaken in long-term hydrated conditions; no biodegradation kinetics or implantation study; strength-porosity trade-off; preparation still requires organic solvents and multistep PCL synthesis.	General regenerative medicine, porous tissue-engineering scaffold, wound dressing, and potentially bone/soft-tissue cryogel, depending on formulation.	[69]

**Table 2:** Laboratory-to-clinic roadmap for PU–CS tissue-engineering scaffolds.

No	Stage	Key Requirement	Critical PU–CS-Specific Issue	Meeting/Not Meeting the Criterion
1	Raw-material characterization	PU-CS composition	PU: molecular architecture; CS: MW, DD, purity;	Reproducible specification
2	Composite formulation	Define PU/CS ratio and processing	Phase separation, hydrogen bonding, morphology	Reproducible physicochemical profile
3	Scaffold characterization	Mechanics, porosity, swelling, degradation	Tissue-specific performance	Predefined acceptance window
4	Biological screening	Cytocompatibility, adhesion	Comparison with PU, CS and controls	No geno—or cytotoxicity + functional benefit
5	Mechanistic validation	Protein adsorption, immune response, biodegradation	Demonstrate mechanism rather than correlation	Reproducible structure–response relationship
6	In vivo proof of concept	Relevant small-animal model	Regeneration vs. scaffold persistence	Functional tissue improvement
7	Long-term/large-animal studies	Clinically relevant properties	Remodeling, immune response, biodegradation products	Safety + durable functional regeneration
8	Manufacturing scale-up	GMP-compatible production	Composition and morphology must remain unchanged	Validated process
9	Sterilization and packaging	Final-device validation	Degradation or altered CS activity	Post-sterilization equivalence
10	Quality control	CQAs and CPPs	Batch-to-batch reproducibility	Validated release criteria
11	Regulatory assessment	Chemical/biological evaluation (ISO)	Composite biodegradation products and classification	Acceptable safety
12	Clinical evaluation	Human safety and efficacy	Clinically meaningful endpoint	Predefined acceptance window

## Data Availability

No new data were created or analyzed in this study. Data sharing does not apply to this article.

## Abbreviations

ALG	alginate
ALP	alkaline phosphatase
APTT	activated partial thromboplastin time
BDI	1,4-diisocyanatobutane
BDO	1,4-butanediol
BHET	bishydroxyethylene terephthalate
DMPA	dimethylol propionic acid
DP	dialdehyde polyurethane
BSA	bovine serum albumin
CA	aminoglucose
CFU	Colony-Forming Unit
CMCS	carboxymethyl chitosan
CNFs	cellulose nanofibers
CNTs	carbon nanotubes
CS	chitosan
CS3K	chito-oligosaccharide (3000 g/mol)/CS30K (30,000 g/mol)/CS30K (300,000 g/mol)
CUR	curcumin
DABCO	triethylenediamine
DBTDL	dibutyltin dilaurate
DD	degree of deacetylation
DDS	drug delivery systems
DMF	N,N-dimethylformamide
DMPA	2,2-dimethylolpropionic acid
DMSO	dimethyl sulfoxide
DOX	doxorubicin
ECM	extracellular matrix
EDA	ethylenediamine
EDC	1-ethyl-3-[3-dimethylaminopropyl]carbodiimide
FA	folic acid
GA	glycolide
hBMSCs	growth, adhesion, and differentiation of human bone marrow mesenchymal stem cells
HDI	1,6-hexamethylene diisocyanate
HTPB	hydroxyl-terminated polybutadiene
HPUC	waterborne polyurethane-chitosan
HUVEC	human umbilical vein endothelial cell
LA	lactide
LDI	lysine diisocyanate
IPDI	isophorone diisocyanate
IPNs	interpenetrating polymer networks
MDI	diphenylmethane-4,4′-diisocyanate
MEK	methyl ethyl ketone
M_n_	number average molar mass
M_w_	weight average molar mass
M_v_	viscosity-average molecular weight
MMT	montmorillonite
MOF	metal–organic framework
MWCNTs	multi-walled carbon nanotubes
NHS	N-hydroxysuccinimide
NIPUs	non-isocyanate polyurethanes
NMP	N-methylpyrrolidone
NPs	nanoparticles
PAA	polyacrylic acid
PCL	poly(ε-caprolactone)
PEG	poly(ethylene glycol)
PET	poly(ethylene terephthalate)
PEO	poly(ethylene oxide)
PGA	poly(glycolic acid)/polyglycolide
P(3HB)	poly(3-hydroxybutyrate)
PLA	poly(lactic acid)/polylactide
PLGA	lactide and glycolide copolymers
PPG	poly(propylene glycol)
PTMC	poly(trimethylene carbonate)
PTMO	poly(tetramethylene oxide)
PU/PUs	polyurethane/polyurethanes
PU–CS	polyurethane–chitosan composites
PUD	polyurethane-diol dispersion
PVA	poly(vinyl alcohol)
SEM	Scanning Electron Microscopy
SnOct_2_	bis(2-ethylhexanoate)
STPP	sodium tripolyphosphate
TEA	triethylamine
TEMPO	2,2,6,6-Tetramethylpiperidine-1-oxyl
TDI	toluene diisocyanate
2,4-TDI	2,4-toluene diisocyanate
2,6-TDI	2,4-toluene diisocyanate
THF	tetrahydrofuran
TIPS	thermally induced phase separation
TMC	trimethylene carbonate
TPP	sodium tripolyphosphate
WPUs	waterborne polyurethanes
